# The TRAIL-Induced Cancer Secretome Promotes a Tumor-Supportive Immune Microenvironment via CCR2

**DOI:** 10.1016/j.molcel.2017.01.021

**Published:** 2017-02-16

**Authors:** Torsten Hartwig, Antonella Montinaro, Silvia von Karstedt, Alexandra Sevko, Silvia Surinova, Ankur Chakravarthy, Lucia Taraborrelli, Peter Draber, Elodie Lafont, Frederick Arce Vargas, Mona A. El-Bahrawy, Sergio A. Quezada, Henning Walczak

**Affiliations:** 1 Centre for Cell Death, Cancer, and Inflammation, Department of Cancer Biology, UCL Cancer Institute, University College London, London WC1E 6DD, UK; 2 Department of Oncology, UCL Cancer Institute, University College London, London WC1E 6DD, UK; 3 Cancer Immunology Unit, Department of Haematology, UCL Cancer Institute, University College London, London WC1E 6DD, UK; 4 Department of Histopathology, Imperial College London, London W12 0NN, UK

**Keywords:** TRAIL, TRAIL-R, FADD, cytokine, CCL2, CCR2, microenvironment, MDSC, tumor

## Abstract

Tumor necrosis factor (TNF)-related apoptosis-inducing ligand (TRAIL) is known for specifically killing cancer cells, whereas in resistant cancers, TRAIL/TRAIL-R can promote metastasis via Rac1 and PI3K. It remains unknown, however, whether and to what extent TRAIL/TRAIL-R signaling in cancer cells can affect the immune microenvironment. Here we show that TRAIL-triggered cytokine secretion from TRAIL-resistant cancer cells is FADD dependent and identify the TRAIL-induced secretome to drive monocyte polarization to myeloid-derived suppressor cells (MDSCs) and M2-like macrophages. TRAIL-R suppression in tumor cells impaired CCL2 production and diminished both lung MDSC presence and tumor growth. In accordance, the receptor of CCL2, CCR2, is required to facilitate increased MDSC presence and tumor growth. Finally, *TRAIL* and *CCL2* are co-regulated with MDSC/M2 markers in lung adenocarcinoma patients. Collectively, endogenous TRAIL/TRAIL-R-mediated CCL2 secretion promotes accumulation of tumor-supportive immune cells in the cancer microenvironment, thereby revealing a tumor-supportive immune-modulatory role of the TRAIL/TRAIL-R system in cancer biology.

## Highlights

• TRAIL induces a cytokine cancer secretome in a FADD- and caspase-8-dependent manner
• FADD promotes tumor growth along with accumulation of M2-like immune cells in vivo
• The TRAIL-induced secretome recruits M2-like immune cells to tumors via CCR2
• TRAIL/CCL2 correlate with a tumor-supportive immune profile in lung cancer patients

## Graphical Abstract

**Figure:**
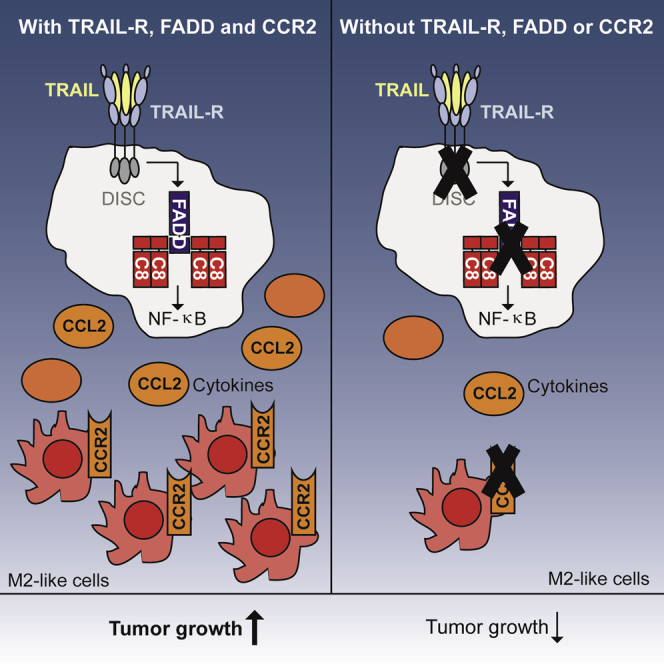

## Teaser

Hartwig et al. show that endogenous TRAIL signaling in cancer cells induces a FADD-dependent secretome that promotes the accumulation of M2-like immune cells and tumor growth via host CCR2.

## Introduction

Chemo- and cytokines are central modulators of the cancer microenvironment, which has been established as one of the hallmark drivers of cancer (Hanahan and Weinberg, 2011). Cancer cells frequently modulate the cytokine milieu to alter the cellular composition of the microenvironment in favor of tumor progression (Hanahan and Weinberg, 2011, Hoesel and Schmid, 2013, Qian et al., 2011). Elevated cytokine levels of interleukin-8 (IL-8), CXCL1, CCL2, and CXCL5 have been associated with increased growth and progression of breast, prostate, and ovarian cancer (Begley et al., 2008, Dong et al., 2013, Fader et al., 2010, Qian et al., 2011, Singh and Lokeshwar, 2009, Zhang et al., 2010). These cytokines can mediate tumor-supportive effects by paracrine recruitment and polarization of cancer-promoting myeloid cell subsets (Chun et al., 2015, Fujita et al., 2011, Highfill et al., 2014). Recently, two alternatively activated myeloid (M2) cell types, CD11b^+^GR1^+^ myeloid-derived suppressor cells (MDSCs) and fully differentiated M2 macrophages, have received particular attention. M2-like cells elicit their tumor-supportive effects by directly promoting tumor growth as well as via immuno-suppression of antitumor effector T cells (Gabrilovich and Nagaraj, 2009, Lesokhin et al., 2012, Mantovani and Sica, 2010, Toh et al., 2011). It is therefore crucial to determine which signals are responsible for creating the cytokine milieu that enables polarization and recruitment of an M2-like immune microenvironment.

Tumor necrosis factor (TNF)-related apoptosis-inducing ligand (TRAIL) is a member of the TNF superfamily (TNF-SF) of cytokines. TRAIL is capable of specifically inducing apoptosis in cancer cells via its death domain (DD)-containing receptors TRAIL-R1 and TRAIL-R2 (also known as DR4 and DR5) (Ashkenazi et al., 1999, Walczak et al., 1999). Although TRAIL’s apoptosis-inducing capacity has been investigated in depth, TRAIL signaling can also induce non-apoptotic, tumor-supporting effects in apoptosis-resistant cancer cells (Johnstone et al., 2008, Lemke et al., 2014b, Newsom-Davis et al., 2009). We recently showed that TRAIL signaling via TRAIL-R2 promotes invasion and metastasis of *KRAS*-mutated cancers by activating Rac1/phosphatidylinositol 3-kinase (PI3K) signaling (von Karstedt et al., 2015). Importantly, this effect was cancer cell autonomous and mediated by the membrane-proximal domain (MPD) of TRAIL-R2, independently of the DD and the apoptosis adaptor protein Fas-associated death domain (FADD). Interestingly, the two DD-containing TRAIL-Rs have previously been linked to the formation of a FADD-containing secondary signaling complex associated with nuclear factor κB (NF-κB) pathway activation, which is known to induce cytokine secretion (Varfolomeev et al., 2005). However, although independent studies have associated the secretion of cytokines with the potential to modulate tumor-associated inflammation, neither TRAIL/TRAIL-R nor FADD-mediated signaling in tumor cells has previously been linked with immuno-regulatory roles in cancer (Balkwill and Mantovani, 2012, Mantovani et al., 2008). We therefore set out to investigate whether the TRAIL/TRAIL-R system influences the tumor immune microenvironment through paracrine cytokine signaling and, if so, to what extent endogenous TRAIL/TRAIL-R-induced modulation of the cancer microenvironment might affect tumor growth.

## Results

### Identification of Cytokines as Functional Drivers within the TRAIL-Induced Secretome

Tumor-secreted factors potently influence cancer biology, and TRAIL has shown the potential to enhance or disable tumor growth depending on the oncogenic context. It is, however, uncharacterized what the TRAIL-induced secretome is constituted of in its entirety. It therefore also remains unexplored what its biological function in cancer might be and, importantly, whether it may contain factors of particular relevance to the described pro-tumorigenic properties of TRAIL. We thus conducted an unbiased proteomic analysis of the TRAIL-induced cancer secretome by mass spectrometry to identify factors that are both secreted upon TRAIL stimulation and functionally enriched according to gene ontology (GO) functional enrichment analysis (Huang et al., 2009).

**Figure 1:**
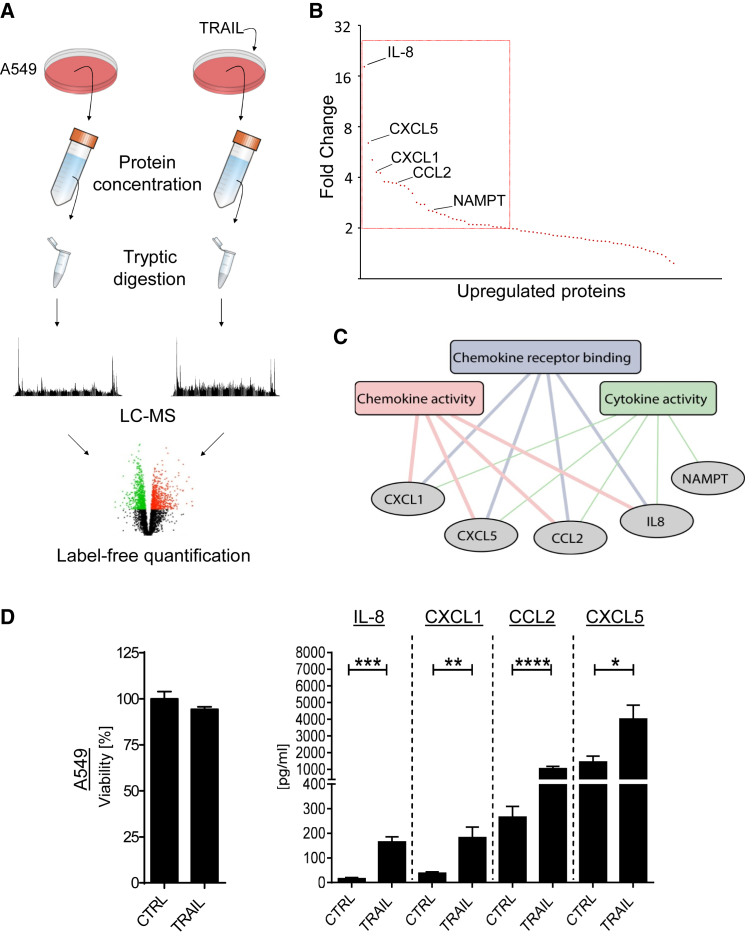
TRAIL Induces a Cytokine Secretome (A) Schematic of the quantitative, label-free secretome profiling approach. (B and C) (B) A pool of all secreted proteins that were upregulated by isoleucine zipper (iz)-TRAIL (100 ng/mL) (>2-fold induction as cutoff) was analyzed by label-free quantification and searched for (C) the most significantly enriched GO molecular functions. Line thickness is representative of the associated p value. Chemokine activity, p = 0.0005; chemokine receptor binding, p = 0.0006; cytokine activity, p = 0.0039. (D) A549 cells were stimulated with iz-TRAIL (100 ng/mL). After 24 hr, cytokine concentrations in the cell supernatants were measured via ELISA; cell viability was determined by CellTiter-Glo. p values were obtained by two-tailed Student’s t test. ^∗^p ≤ 0.05, ^∗∗^p < 0.01, ^∗∗∗^p < 0.001, ^∗∗∗∗^p < 0.0001. Data are presented as mean ± SEM, n = 3. See also Figure S1.

Utilizing a label-free quantitative workflow (Figure 1A), we detected a total of 1,723 proteins (Table S1, available online). These were then quantified and used for statistical testing of the differential abundance between TRAIL-stimulated and unstimulated samples. Of the 1,723 detected proteins, 720 were classified as belonging to “the secretome” because they were defined as secreted or membrane proteins according to the UniProt database and/or predicted by the Phobius transmembrane topology and signal peptide predictor (Käll et al., 2004). A pool of 35 of these proteins, which were upregulated greater than 2-fold by TRAIL (Figure 1B; Table S1), were subsequently analyzed for the most significantly enriched molecular functions, as defined by GO terms, using the functional annotation tool Database for Annotation, Visualization, and Integrated Discovery (DAVID). This analysis revealed functional enrichment of CXCL1, CXCL5, CCL2, IL-8, and NAMPT (Figure 1C). Moreover, CXCL1, CXCL5, CCL2, and IL-8 were highly induced, as determined by label-free quantification, and the only constituents identified in the secretome that continuously clustered together in three separate GO molecular functions, indicating a high potential for functional significance (Figures 1B and 1C). In line with their high potential for functional impact, CXCL1, CXCL5, CCL2, and IL-8 were also significantly induced by TRAIL in different cancer cell lines, as determined by ELISA, antibody-based cytokine array, and qPCR (Figures 1D and S1A–S1C).

We recently showed that tumor cell-expressed TRAIL and TRAIL-R2 promote cancer progression, invasion, and metastasis by cancer cell-autonomous activation of Rac1 via the MPD of TRAIL-R2 independently of FADD (von Karstedt et al., 2015). We therefore next tested whether this previously identified cell-autonomous migration pathway involved cytokine production from cancer cells. This was, however, not the case because conditioned supernatants of wild-type (WT) A549 cells were incapable of rescuing the impaired migration of TRAIL-R2 knockdown (KD) cells (Figure S1D). Conversely, cytokine induction by TRAIL was also independent of Rac1 signaling because Rac1 inhibition did not block TRAIL-mediated cytokine induction (Figure S1E). Last, re-expression of a C-terminally truncated TRAIL-R2 containing only the migration-inducing MPD was not capable of inducing cytokine secretion, whereas already low levels of re-expressed full-length TRAIL-R2 containing the DD were sufficient to re-instate TRAIL-induced chemokine production (Figure S1F). Together, these results demonstrate that TRAIL must induce its secretome via a distinct mechanism.

### Surviving Cancer Cells Are the Source of TRAIL-Induced Cytokines

CD95L-induced apoptosis has recently been associated with cytokine release from dying cells as a means of generating “find me signals” that assure rapid removal of dead cells (Cullen et al., 2013).

**Figure 2:**
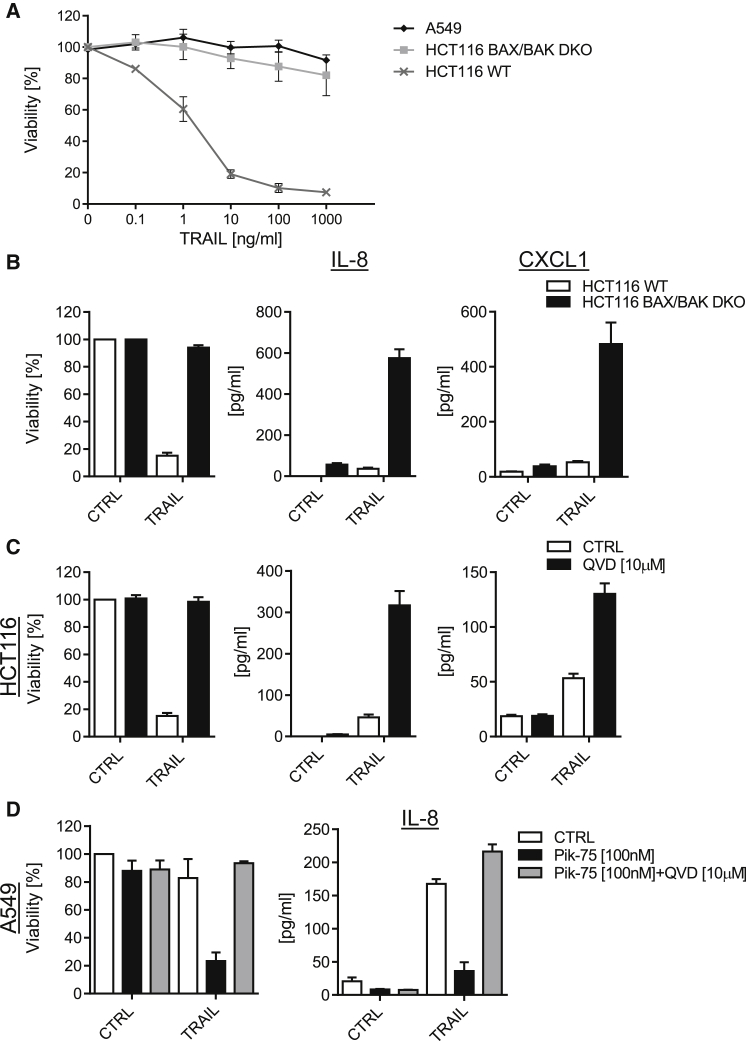
Surviving Cancer Cells Produce Cytokines upon TRAIL Stimulation (A) A549, HCT116 WT, and HCT116 BAX/BAK KO cells were stimulated with the indicated concentrations of iz-TRAIL for 24 hr; cell viability was determined by CellTiter-Glo. (B) HCT116 WT or HCT116-BAX/BAK KO cells were stimulated with iz-TRAIL (100 ng/mL) for 24 hr; cell viability was determined by CellTiter-Glo and cytokine concentrations in the cell supernatants were measured by ELISA. (C) HCT116 WT cells were pre-incubated with QVD (10 μM) or DMSO for 30 min, followed by addition of iz-TRAIL (100 ng/mL for 24 hr; cell viability and cytokine concentrations were determined as above. (D) A549 cells were pre-incubated with PIK-75 (100 nM) or QVD (10 μM) + PIK-75 (100 nM) for 30 min, followed by addition of iz-TRAIL (100 ng/mL); cell viability and IL-8 concentration were determined as above. Data are presented as mean ± SEM, n = 3. See also Figure S2.

We therefore determined whether cytokines were actively secreted by surviving cells or passively released from dying cells upon TRAIL treatment. To do so, we utilized TRAIL-sensitive HeLa and HCT116 WT cells compared with TRAIL-resistant isogenic HCT116 BAX/BAK knockout (KO) and A549 cells (Figures 2A and S2A). Killing doses of TRAIL induced very low levels of IL-8 and CXCL1 in HCT116 WT cells (Figure 2B), whereas stimulation of TRAIL-resistant HCT116 BAX/BAK KO cells led to very robust secretion of IL-8 and CXCL1. In addition, blocking cell death with the pan-caspase inhibitor Q-VD-OPh (QVD) rescued cytokine induction in HCT116 WT and HeLa cells (Figures 2C and S2A). Importantly, caspase activity did not affect cytokine induction independently of its effect on cell death (Figures S2A and S2B). Supporting a central role for live cells in the production of cytokines, sensitizing A549 cells to cell death using the small-molecule inhibitor PIK-75, which enhances TRAIL-induced apoptosis via the inhibition of CDK9 and consequent downregulation of cFLIP and Mcl-1 (Lemke et al., 2014a), drastically decreased TRAIL-mediated IL-8 induction as fewer live cells were present to produce IL-8. Cell viability and cytokine production could again be rescued by co-treatment with QVD (Figure 2D). Collectively, these results show that cells surviving TRAIL stimulation are the main source of TRAIL-induced cytokine secretion, which is independent of caspase activity.

### Caspase-8 and FADD Are Required for TRAIL-Induced Cytokine Secretion

**Figure 3:**
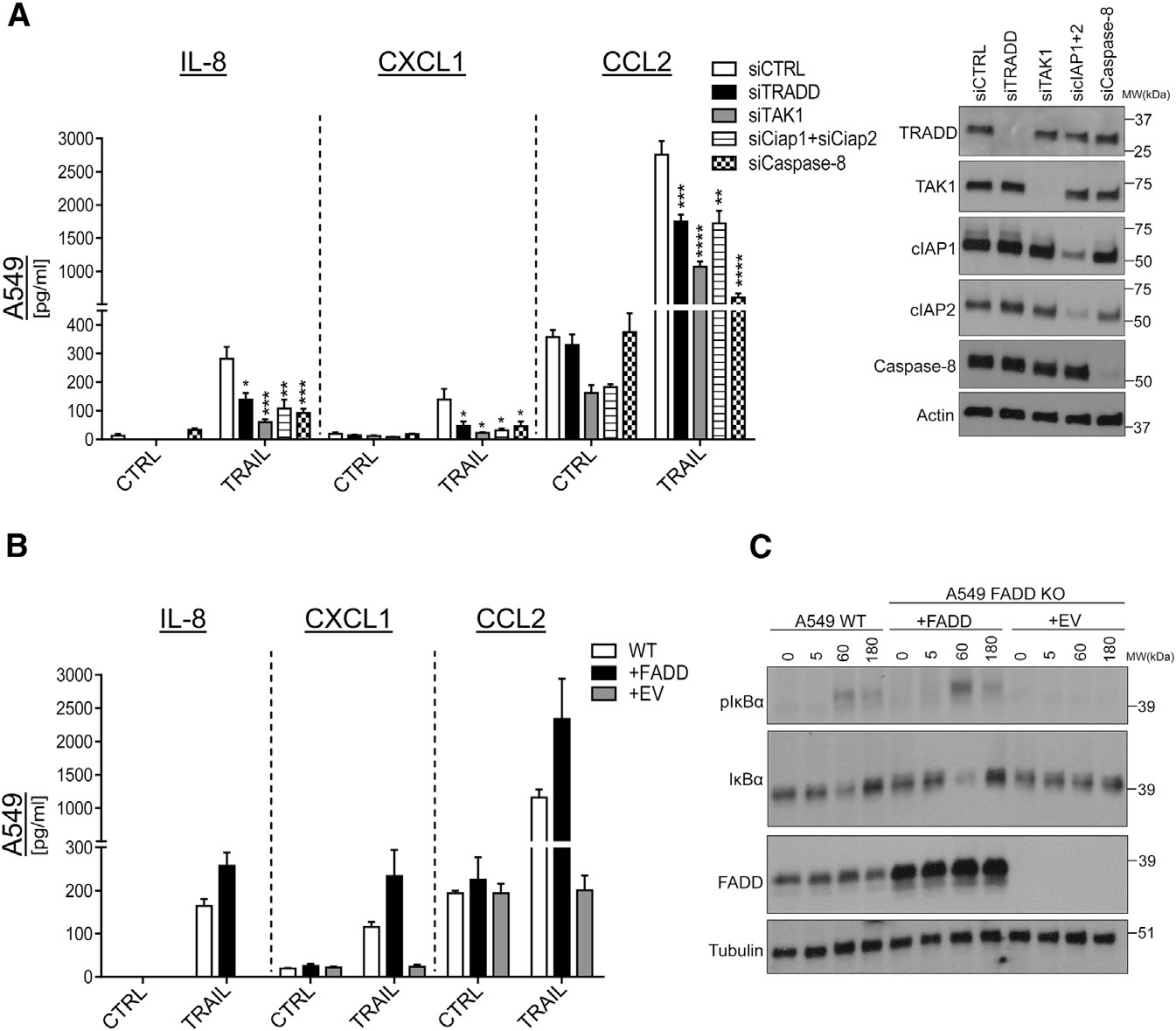
Caspase-8 and FADD Presence Is Required for TRAIL-Mediated Cytokine Induction (A) A549 WT cells were transiently transfected with small interfering RNA (siRNA) against the indicated targets for 48 hr and stimulated with QVD (10 μM) (CTRL) or QVD (10 μM) + iz-TRAIL (100 ng/mL); 24 hr later, cytokine concentrations were determined by ELISA. A representative western blot is shown. (B) A549 WT or FADD KO, either empty vector (+EV) or FADD (+FADD) reconstituted, were stimulated with iz-TRAIL (100 ng/mL) for 24 hr, followed by determination of cytokine concentrations by ELISA. (C) Cells as in (B) were stimulated with iz-TRAIL (100 ng/mL) for the indicated times, followed by immunoblotting for the indicated proteins. Data are presented as mean ± SEM, n ≥ 3. A representative experiment of n = 2 is shown. See also Figures S3 and S4.

We next addressed the signaling requirements for TRAIL-induced cytokine production. Because caspase activity was not required, we first sought to exclude an activity-independent role for caspase-8. Surprisingly, KD of caspase-8 reduced TRAIL-mediated secretion of IL-8, CXCL1, and CCL2 (Figures 3A, S3A, and S3B). Thus, TRAIL-mediated cytokine induction requires the presence of caspase-8, but not its activity (Figure S2B).

Because recruitment of caspase-8 to the death-inducing signaling complex (DISC) depends on the adaptor molecule FADD, we next assessed the effect of FADD absence on cytokine production. In line with a requirement for caspase-8, FADD deficiency entirely abrogated TRAIL-induced cytokine secretion in human and murine lung cancer cells (Figures 3B, S3C, and S3D). TRAIL-R surface expression levels were comparable between WT, control (CTRL), and FADD-deficient cells (Figure S3E). FADD deficiency did not sensitize these cells to necroptosis because neither A549 nor murine Lewis lung carcinoma (3LL) cells express detectable levels of RIPK3 (Figure S3F). In accord, neither cell line could be sensitized to necroptosis by addition of second mitochondria-derived activator of caspases (SMAC) mimetics and zVAD (Figure S3G). Importantly, the secretion of all discovered cytokines was reinstated by reconstitution of FADD expression (Figures 3B and S3H).

Downstream of FADD and the DISC, a secondary complex has been associated with TRAIL-mediated gene-activatory signaling (Varfolomeev et al., 2005). Here the role of TRADD, a crucial adaptor in TNFR1 signaling, has remained controversial, whereas its role in TNF-induced gene activation is well established (Pobezinskaya and Liu, 2012, Hsu et al., 1995). To explore whether TRADD might serve as an adaptor in the secondary complex, we silenced TRADD and determined how this affected TRAIL-mediated cytokine secretion (Figures 3A, S3A, and S3B). Interestingly, cytokine production was significantly decreased in TRADD-silenced A549 and 3LL cells, identifying TRADD as required for this TRAIL signaling output.

We therefore next tested how the ablation of expression of several other factors that are also involved in TNFR1 signaling (Mahoney et al., 2008) affected TRAIL-induced cytokine expression. In support of a promoting role of cIAP1/2 in TRAIL-mediated cytokine secretion, both RNAi-mediated silencing of cIAP1/2 as well as their pharmacological inhibition with the SMAC mimetic compound SM-83 (Lecis et al., 2013) decreased the levels of TRAIL-induced IL-8, CXCL1, and CCL2 (Figures 3A, S3I, and S3J). In addition, silencing of RIPK1 also suppressed TRAIL-induced cytokine secretion (Figure S3B). KD of TAK1, a kinase crucial for IκB kinase (IKK) activation and gene activation in TNFR1 signaling (Wang et al., 2001), decreased TRAIL-induced cytokine secretion. This effect was attributable to TAK1 kinase activity because specific TAK1 kinase inhibition exerted similar effects (Figures 3A, S3A, S4A, and S4B).

Because absence of TRADD, cIAP1/2, or TAK1, all known inducers of TNFR1 gene-activatory signaling, phenocopied FADD deficiency regarding TRAIL’s cytokine induction, we next tested whether FADD deficiency would affect TRAIL-induced gene-activatory signaling. Indeed, FADD deficiency abolished IκB phosphorylation upon TRAIL treatment, which could be reinstated by FADD reconstitution (Figure 3C), demonstrating a requirement of FADD for TRAIL-induced gene activation. Accordingly, small-molecule inhibition of IκB phosphorylation also abrogated cytokine secretion (Figures S4C and S4D). Of note, CD95L also induced FADD- and caspase-8-dependent cytokine secretion in A549 cells but failed to do so in murine 3LL cells (Figures S4E and S4F) even though the CD95L we used was able to kill mouse embryonic fibroblasts (MEFs) (Figure S4G). The lack of CD95L-mediated cytokine induction in 3LL cells was most likely due to absent CD95 surface expression (Figure S4H). By contrast, TNF-mediated cytokine induction required neither FADD nor caspase-8 (Figures S4E and S4F). Thus, caspase-8 and FADD are required for cytokine secretion by the FADD-recruiting TRAIL/TRAIL-R and CD95L/CD95 systems, but, interestingly, not for cytokine induction by TNFR1.

### FADD Promotes Tumor Growth and Accumulation of Alternatively Activated Myeloid Cells

**Figure 4:**
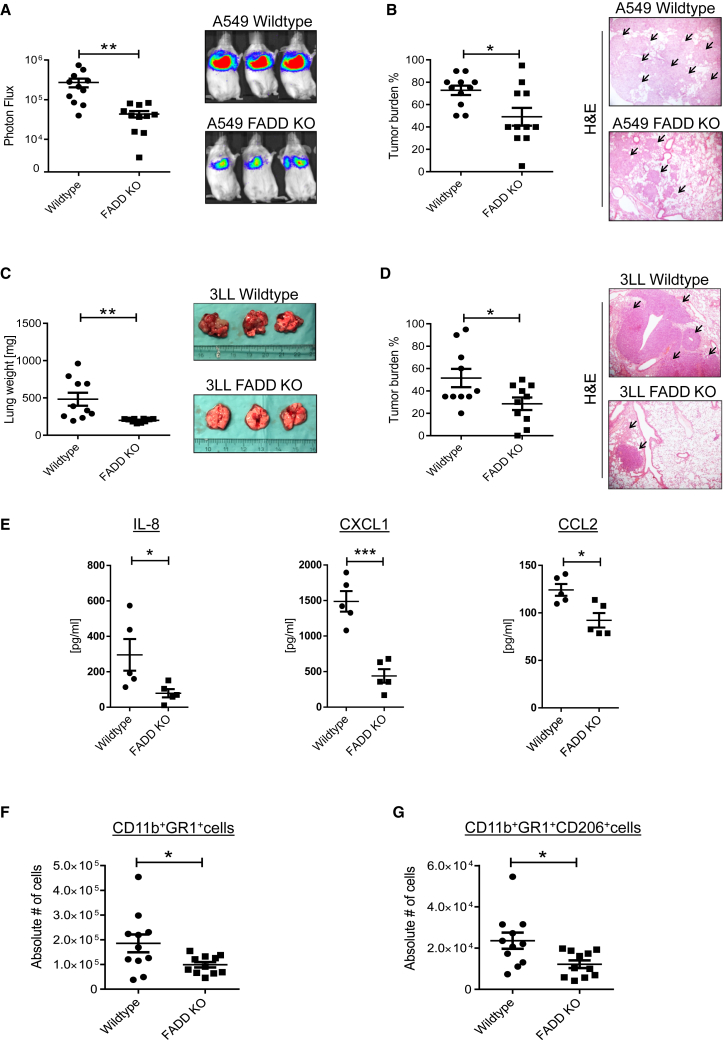
FADD Promotes Tumor Growth and Accumulation of Alternatively Activated Myeloid Cells (A) Severe combined immunodeficiency (SCID)/beige mice were injected with 2 × 10^6^ A549 WT or FADD KO cells stably expressing luciferase into the lateral tail vein. Tumor burden was assessed after 24 days via bioluminescence imaging. n = 11/group. Representative images are shown. (B) Histological quantification of tumor burden. Representative images of H&E-stained lung sections (5× magnification) are shown. (C) C57BL/6 mice were injected with 5 × 10^5^ 3LL cells into the lateral tail vein. Lung weights were determined 28 days later. Representative lungs are shown. (D) Histological quantification of tumor burden in lungs from mice shown in (C). Representative images of H&E-stained lung sections (5× magnification) are shown. (E) The indicated cytokines were quantified in lung homogenates by ELISA. (F and G) Absolute number of (F) CD11b^+^Gr1^+^ or (G) CD11b^+^Gr1^+^CD206^+^ cells within tumor-bearing lungs. Unpaired, two-tailed Student’s t test was performed to determine significance. ^∗^p ≤ 0.05, ^∗∗^p < 0.01, ^∗∗∗^p < 0.001. Data are represented as mean ± SEM. See also Figure S5.

Although evidence from non-small-cell lung cancer (NSCLC) patients indicates that high *FADD* mRNA expression correlates with a poor survival prognosis (Chen et al., 2005), mechanistic insight into this correlation is lacking. Based on these reports and our observed requirement of FADD for TRAIL-mediated cytokine induction, we next investigated whether cancer cell-expressed FADD would affect tumor growth in vivo. Strikingly, deletion of human FADD in an orthotopic mouse model of NSCLC strongly diminished lung tumor burden (Figures 4A, 4B, S5A, and S5B). Importantly, this effect was recapitulated in a syngeneic model wherein deletion of murine FADD in two independent 3LL clones significantly impaired tumor growth, demonstrating a tumor-promoting role of FADD across species (Figures 4C, 4D, and S5C). Of note, FADD deficiency did not affect proliferation in vitro (Figure S5D).

The fact that the presence of FADD in tumor cells enhances cancer cell growth in vivo, but not in vitro, suggested that FADD might favor tumor growth by enabling an interaction with the tumor microenvironment. We therefore quantified the concentration of human cytokines in murine lungs and found that levels of IL-8, CXCL1, and CCL2, which our in vitro analysis had identified as being induced by TRAIL in an FADD-dependent manner (Figure 3B), were decreased in lungs containing FADD-deficient tumors (Figure 4E). Since these cytokines were previously reported to be associated with the influx of GR1^+^ cells (Highfill et al., 2014, Toh et al., 2011), we compared myeloid immune cell infiltration in the microenvironment of FADD-proficient and FADD-deficient tumors. Importantly, FADD-deficient tumors contained significantly fewer infiltrating CD11b^+^GR1^+^ cells with lower CD206^+^ expression (Figures 4F, 4G, S5E, and S6H), whereas the overall levels of total CD45^+^ cells were comparable between the two groups (Figure S5F). Expression of CD11b, GR1, and CD206 has been associated with alternatively activated M2-like myeloid cells that can elicit tumor-supportive functions (Gabrilovich and Nagaraj, 2009, Lesokhin et al., 2012). Therefore, FADD presence promotes the growth of lung tumors, encourages the formation of a tumor-supportive cytokine milieu, and increases the accumulation of M2-like myeloid cells.

### The TRAIL-Induced Secretome Polarizes Monocytes to M2-like Cells

**Figure 5:**
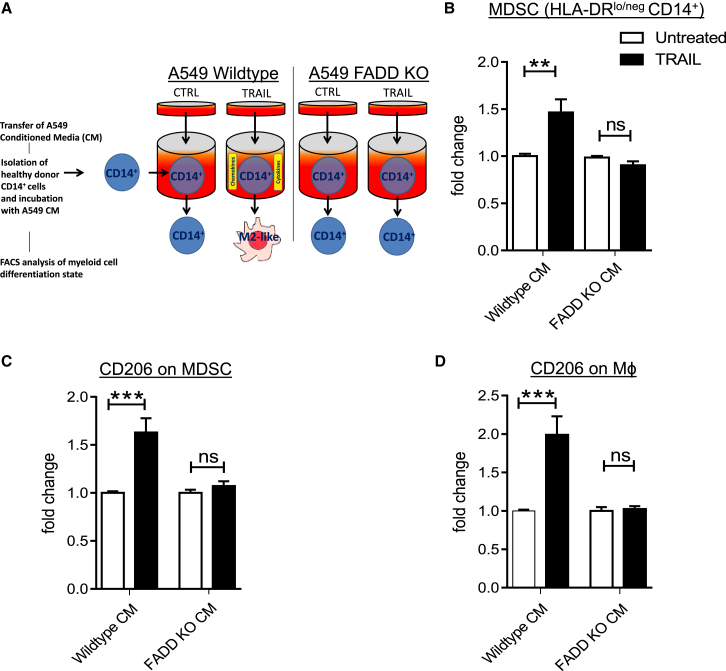
A TRAIL-Induced Secretome Polarizes Human Monocytes to Alternatively Activated Myeloid Cells (A) Schematic of the monocyte polarization protocol. (B–D) CD14^+^ cells were isolated from healthy donor peripheral blood mononuclear cells (PBMCs) via magnetic CD14^+^ microbeads and incubated with untreated or iz-TRAIL-treated (100 ng/mL) WT or FADD KO A549 conditioned medium (CM). After 48 hr, treated myeloid cells were stained with fluorochrome-labeled antibodies against HLA-DR, CD14, and CD206 and analyzed by flow cytometry. Data are presented as fold change in HLA-DR^lo/neg^ CD14^+^ cells (B), HLA-DR^lo/neg^, CD14^+^, CD206^+^ cells (C), or HLA-DR^+^, CD14^+^ CD206^+^ macrophages (D) upon iz-TRAIL-induced CM incubation. Unpaired, two-tailed Student’s t test was performed to determine significance. ns = p > 0.05, ^∗∗^p < 0.01, ^∗∗∗^p < 0.001. Data are presented as mean ± SEM, n = 4.

So far, our results established FADD presence in tumor cells as a significant driver of both in vivo cytokine production and the presence of alternatively activated myeloid cells. Because we found TRAIL to induce the very same cytokines in a FADD-dependent manner, we next investigated whether the TRAIL-induced FADD-dependent secretome might influence immune cell polarization. To this end, human healthy donor CD14^+^ cells were cultured with supernatants of CTRL or TRAIL-treated A549 WT or FADD KO cells (Figure 5A). Strikingly, supernatants of WT A549 cells treated with TRAIL polarized healthy donor CD14^+^ cells toward an HLA-DR^lo/neg^ phenotype, an immune cell population equivalent to murine CD11b^+^GR1^+^ cells (Sevko and Umansky, 2013) that we observed in vivo (Figures 4F and 5B). Furthermore, HLA-DR^lo/neg^ as well as HLA-DR^+^ cells displayed increased levels of CD206 expression, indicating polarization toward MDSC and fully differentiated M2 macrophage phenotypes, respectively (Figures 5C and 5D). Therefore, TRAIL can trigger the secretion of myeloid cell-polarizing factors from tumor cells in a FADD-dependent manner.

### Cancer Cell-Expressed TRAIL-R Supports Tumor Growth and Recruitment of Tumor-Supportive Infiltrates in a Host CCR2-Dependent Manner

As the full extent of the effect of cancer cell-expressed TRAIL-R on immune cell infiltration can most suitably be assessed in immune-proficient mice, we next made use of the established orthotopic 3LL model. Importantly, all crucial factors identified in the TRAIL-induced secretome were also induced by murine TRAIL in 3LL cells, with CCL2 being produced at the highest level (Figure S6A). CCL2 was recently identified as a crucial chemoattractant cytokine for alternatively activated myeloid cells via its receptor, CCR2 (Chun et al., 2015). In line with this, CCL2 expression has also been shown to polarize human peripheral blood CD11b^+^ cells toward an anti-inflammatory CD206^+^ M2-phenotype (Roca et al., 2009, Sierra-Filardi et al., 2014). Because our proteomic analysis identified CCL2 to be among the TRAIL-induced factors with a high potential for biological functionality, we hypothesized that stimulation of cancer cell-expressed TRAIL-R may result in CCL2 production by tumor cells and, thereby, facilitate the accumulation of alternatively activated M2-type cells in the tumor microenvironment.

**Figure 6:**
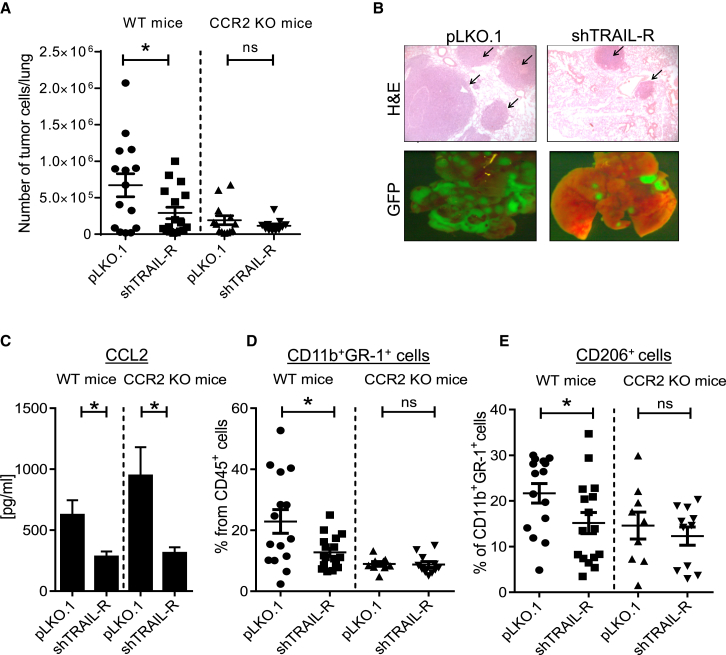
Cancer Cell-Expressed TRAIL-R Supports Tumor Growth and Recruitment of Tumor-Supportive Infiltrates in a Host CCR2-Dependent Manner (A) WT or CCR2 KO mice were injected with 5 × 10^5^ 3LL-GFP empty vector (pLKO.1) or sh-containing vector for TRAIL-R (shTRAIL-R) cells via the lateral tail vein and left to develop tumors for 28 days. Upon dissociation of lungs, the absolute number of tumor cells was determined by measuring GFP via fluorescence-activated cell sorting (FACS). WT mice pLKO.1, n = 15; shTRAIL-R, n = 17; CCR2 KO mice pLKO, n = 13; shTRAIL-R, n = 11. (B) Top: H&E staining of fixed lungs (5× magnification). Bottom: GFP-positive 3LL-containing lungs as determined by bright-field microscopy (1.5× magnification). (C) CCL2 protein levels in lung homogenates from WT mice measured by ELISA. (D and E) Dissociated lungs were stained with fluorochrome-labeled antibodies for (D) CD11b^+^GR-1^+^ or (E) CD11b^+^GR-1^+^CD206^+^ cells and analyzed by FACS. Data are represented as mean ± SEM. Unpaired, two-tailed Student’s t test was performed to determine significance. ns = p > 0.05, ^∗^p ≤ 0.05. See also Figure S6.

To test this hypothesis, we prepared stable TRAIL-R KD (shTRAIL-R) or CTRL (pLKO.1) 3LL cells co-expressing GFP at similar levels (Figure S6B). These cells were subsequently injected into WT or CCR2-deficient mice (Figure S6B). Determination of GFP positivity in tumor-bearing lungs and histological quantification revealed a substantially lower tumor burden in WT mice injected with shTRAIL-R 3LL (Figures 6A and 6B). This was confirmed using different TRAIL-R-specific small hairpin RNAs (shRNAs), excluding off-target effects as causative for the observed result (Figure S6C). In vitro cell growth was similar in pLKO.1 and shTRAIL-R cells (Figure S6D). Importantly, the difference in tumor burden between pLKO.1 and shTRAIL-R tumors was completely absent when the host was devoid of CCR2 (Figure 6A). Moreover, the decreased tumor burden in mice injected with shTRAIL-R cells significantly correlated with decreased CCL2 protein and transcript levels in shTRAIL-R cell-containing lungs (Figures 6C and S6E). Interestingly, the indicated difference in CCL2 protein levels was also apparent in CCR2 KO mice, showing that it is not the mere extent of tumor burden that determines CCL2 amounts but, instead, the ability of tumor cells to produce CCL2, a capacity that is impaired in the absence of TRAIL-R (Figure 6C). These results provide in vivo evidence that expression of TRAIL-R by tumor cells is required for their production of CCL2, which, in turn, mediates pro-tumorigenic effects via CCR2 expressed on host cells.

In line with a functional involvement of alternatively activated myeloid cells in influencing tumor burden, the infiltration of pLKO.1 versus shTRAIL-R 3LL tumors was significantly decreased by shTRAIL-R in WT, but not in CCR2-deficient, mice (Figures 6D and 6E). Thus, in the absence of host CCR2, tumor cell-derived CCL2 that is induced downstream of cancer cell-expressed TRAIL-R (Figure 6C) fails to promote the presence of alternatively activated myeloid cells in the tumor microenvironment, coincident with diminished tumor growth. In line with our results implicating FADD as crucial for TRAIL-R-mediated CCL2 secretion, 3LL FADD KO cells replicated our results observed in CCR2 KO mice regarding tumor burden and myeloid infiltrates (Figures S6G and S6H). Because 3LL cells failed to secrete cytokines in response to CD95L in vitro (Figures S4F–S4H), we conclude that endogenous TRAIL/TRAIL-R induces secretion of CCL2 from tumor cells in a FADD-dependent manner and that this CCL2 is required to facilitate the accumulation of tumor-promoting myeloid cells in vivo.

### *TRAIL* and *CCL2* Correlate with a Tumor-Supportive Immune Profile in Lung Adenocarcinoma Patients

**Figure 7:**
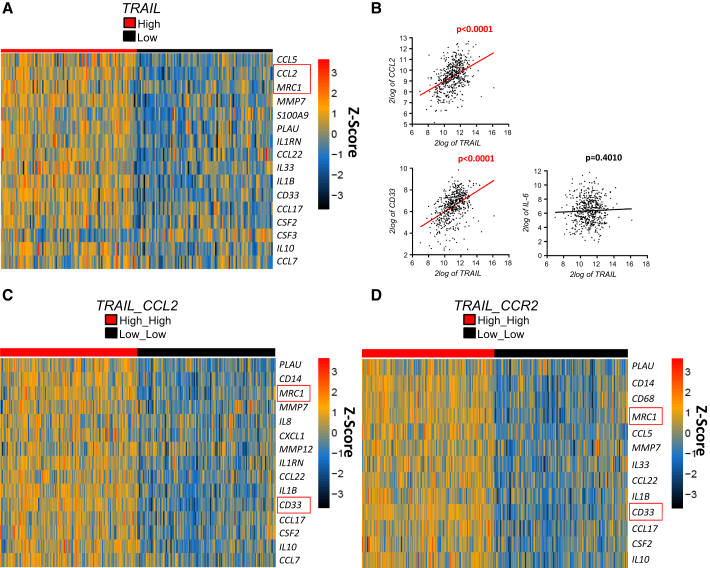
*TRAIL* and *CCL2* Correlate with a Tumor-Supportive Immune Profile in Lung Adenocarcinoma Patients (A–D) RNA-seq expression data from human lung adenocarcinoma biopsy samples (n = 489) were analyzed for association of *TRAIL* (*TNFSF10*)/*CCL2*/*CCR2* expression for a curated list of immune-related genes. (A) Heatmap of genes significantly co-expressed (p = 0.01) with *TRAIL*, showing log2 expression *Z* scores for 20% of samples with highest or lowest *TRAIL* expression. (B) Correlation data for *TRAIL* versus *CCL2*, *CD33*, or *IL-6* expression. The statistical significance of correlations was determined using Pearson’s correlation coefficient. The linear regression curve is shown as a red or black line for significant or non-significant correlations, respectively. (C) As in (A) for 50% of samples with highest or lowest composite *TRAIL* and *CCL2* expression. (D) As in (C) for samples with highest or lowest composite *TRAIL* and *CCR2* expression. See also Figure S7.

To evaluate whether TRAIL’s pro-cancer immunomodulatory role would be reflected in cancer patient-derived gene expression data, we subjected RNA sequencing (RNA-seq) data from a cohort of 489 lung adenocarcinoma (LUAD) patients (Cancer Genome Atlas Research Network, 2014), obtained from the Cancer Genome Atlas (TCGA) (https://cancergenome.nih.gov/), to bioinformatic coexpression analysis focusing on immune cell markers and cytokines (Table S2). Strikingly, *TRAIL* expression showed a significant positive correlation with the expression of 16 M2 myeloid cell markers and cytokines associated with their expansion (Figures 7A and 7B). Importantly, this included *CCL2* as well as *CD206* (*MRC1*), whose murine equivalents we found to be upregulated in mice dependent on the endogenous TRAIL/TRAIL-R system. In line with this, expression of *IL-6*, which we found not to be regulated by TRAIL in lung cancer cells, also did not correlate with *TRAIL* expression in patients (Figure 7B). However, interestingly, many factors correlating with *TRAIL* also correlated with *IL-6* (Figure S7A). Since not all patients expressing high *TRAIL* levels expressed low *IL-6* levels or vice versa (Figure S7B), the possibility that these two cytokines might replace each other in inducing M2-like infiltrates can be excluded. Therefore, these data support that TRAIL does not directly induce IL-6 but suggest that both cytokines might form part of a co-regulated cytokine network in lung adenocarcinoma.

Our data obtained in mice, together with these human data, suggest that a TRAIL/CCL2 axis might also be involved in modulating the human tumor immune microenvironment. We therefore next analyzed composite *TRAIL* and *CCL2* high versus low expression levels in respect to co-expression with factors involved in M2-like cell signaling. In line with a decisive role for TRAIL-induced CCL2 in generating an alternatively activated immune microenvironment, the expression levels of 15 M2 markers and cytokines significantly correlated with composite *TRAIL*/*CCL2* levels (Figure 7C).

Because TRAIL/TRAIL-R-induced CCL2 elicited its tumor-supportive effect via CCR2, we also determined whether, and if so, which, immune cell markers and cytokines were co-regulated with composite *TRAIL*/*CCR2* expression. Here, 13 factors were identified to significantly correlate, providing a strikingly similar expression profile as before, with ten of these overlapping with factors associated with *TRAIL*/*CCL2* expression (Figure 7D). Again, *CD206* (*MRC1*) formed part of this group, indicating its association with *TRAIL*/*CCL2* as well as with *TRAIL*/*CCR2* and thereby revealing a potential connection between TRAIL, CCL2, and CCR2 in promoting M2-like myeloid cell accumulation within human tumors. Together, these data implicate endogenous TRAIL with an increase in tumor-supportive cytokines as well as M2-myeloid markers, thereby extending the relevance of our findings to lung adenocarcinoma patients.

## Discussion

Treatment with TRAIL is capable of specifically killing cancer cells without harming non-transformed cells (Ashkenazi et al., 1999, Walczak et al., 1999). However, some cancer cells upregulate TRAIL-R expression, and we recently showed that this facilitates progression of *KRAS*-mutated cancers via cancer cell-autonomous Rac1 activation independently of FADD (von Karstedt et al., 2015). Here we identify a distinct, additional tumor-supportive function of TRAIL-R signaling in cancer cells that requires FADD. Moreover, we show that endogenous TRAIL induces the FADD-dependent secretion of cytokines, most importantly CCL2, resulting in polarization of myeloid cells toward M2-like cells and the accumulation of such alternatively activated myeloid cells in the tumor microenvironment in a CCL2/CCR2-dependent manner, thereby contributing to tumor growth.

Expression of TRAIL-R1 and/or TRAIL-R2, together with their adaptor protein FADD, is known to be essential for induction of TRAIL-induced apoptosis. Paradoxically, both receptors and FADD have recently been independently associated with promoting NF-κB induction and tumor growth, whereas a mechanistic background concerning the involvement of FADD, in particular regarding its effect on the microenvironment, has been lacking (Bowman et al., 2015, Tang et al., 2009, Trauzold et al., 2006). Although cell-autonomous migration of *KRAS*-mutated cells was specifically mediated via the MPD of TRAIL-R2 (von Karstedt et al., 2015), we show here that the induction of cytokines by TRAIL does not depend on the MPD but, instead, requires the DD-mediated recruitment of FADD. Because TRAIL-R1 as well as TRAIL-R2 express a highly conserved DD, it is conceivable that both of these receptors are involved in TRAIL-induced cytokine production. In line with this, stable KD of either TRAIL-R1 or TRAIL-R2 significantly reduced TRAIL’s cytokine induction (data not shown). Therefore, TRAIL/TRAIL-R-mediated, FADD-dependent cytokine induction represents a distinct cancer-promoting function of this ligand-receptor system. Because FADD absence did not affect TNF-mediated cytokine secretion, and CD95L did not induce the secretion of cytokines from 3LL cells, it is unlikely that these death ligands are responsible for the decreased tumor burden observed in the syngeneic FADD-deficient 3LL model.

As we find that TRAIL does not only induce cytokines in lung cancer cell lines but also in colorectal and pancreatic cancer lines, it is likely that the principal mechanism of a TRAIL-generated, tumor-supportive immune environment may also apply to other cancer types. However, we cannot exclude that, in certain cancers, other death ligands may facilitate the accumulation of tumor-supportive immune cells via a similar mechanism as we have shown for TRAIL. Indeed, our in vitro findings with CD95L suggest that this may be the case and that, in certain cancers, TRAIL and CD95L possibly cooperate in creating a tumor-supportive immune microenviroment.

Although FADD is a crucial mediator of apoptosis, it has been implicated in facilitating tumor promotion in hepatocellular carcinoma (HCC) and is associated with a poor clinical outcome in head and neck cancer and NSCLC patients (Bowman et al., 2015, Ehlken et al., 2014, Rasamny et al., 2012). In NEMO-deficiency-driven HCC, FADD deletion was shown to rescue from aberrant apoptosis, hepatitis, and carcinoma development independently of TRAIL-R. In contrast to HCC, lung cancer is commonly driven by NF-κB signaling, resulting in cytokine induction, whereas in this model of HCC, abrogation of NF-κB signaling through NEMO deletion results in inflammatory cell death, which causes cancer progression (Luedde et al., 2007, Maeda et al., 2005, Meylan et al., 2009), implying divergent cancer etiologies for the two models. Analysis of how these discrepancies may result in opposing roles of TRAIL-R will be interesting to investigate in the future.

Evidence from NSCLC patients indicates that high *FADD* mRNA expression correlates with a poor survival prognosis (Chen et al., 2005). In fact, FADD has recently been shown to promote lung cancer progression in a KRAS-driven, genetically engineered mouse model (Bowman et al., 2015). Interestingly, cancer cell-specific FADD deletion was also associated with decreased myeloid infiltrates. However, a causative role of FADD regarding immune infiltrates was not investigated. It is therefore likely that TRAIL and its function as a promoter of a tumor-supportive immune microenvironment may also play a role in this *KRAS*-induced genetic model. Our data further demonstrate that endogenous TRAIL/TRAIL-R-mediated production of cytokines by tumor cells contributes to the polarization of the lung microenvironment toward increased tumor-supportive, alternatively activated myeloid infiltrates. We identify endogenous TRAIL-R-induced CCL2 and its activity on host-derived CCR2-expressing cells as crucial for the formation of a tumor-supportive myeloid compartment because TRAIL-R-dependent differences in tumor burden were observed in WT, but not in CCR2-deficient, mice.

Although these experiments identify host cell expression of CCR2 and tumor cell expression of TRAIL-R and CCL2 as required for this pro-tumorigenic crosstalk between the TRAIL/TRAIL-R and CCL2/CCR2 systems, the source for TRAIL can be many fold. In the case of the A549 model, the tumor cells themselves could serve as the source because silencing of endogenous TRAIL reduces their cytokine secretion (data not shown). However, many normal cell types, including various different immune cells such as monocytes, T cells, natural killer cells, and dendritic cells, have been shown to express TRAIL (Fanger et al., 1999, Griffith et al., 1999, Kayagaki et al., 1999, Mariani and Krammer, 1998, Mirandola et al., 2004) and could therefore contribute to tumor promotion by TRAIL and possibly even provide a positive feedback loop. Interestingly, and in line with a role for TRAIL-induced cytokines in affecting the myeloid cell compartment, the supernatant of TRAIL-treated HT1080 cells has previously been found to attract human macrophages in vitro (Varfolomeev et al., 2005). Here we identify that within the TRAIL secretome, CCL2 fulfils a central function in the formation of a tumor-supportive myeloid compartment that is achieved in vivo via the engagement of CCR2 on host cells.

Although CCL2 is described as the principal endogenous ligand in humans and mice, CCR2 also binds CCL7, CCL8, and CCL11 (El Khoury et al., 2007, Naert and Rivest, 2013). Importantly, however, CCL2, whose main receptor is CCR2, was the only CCR2 ligand we found to be induced by the TRAIL/TRAIL-R system (data not shown). This implicates CCL2 as the CCR2 ligand that is induced by endogenous TRAIL and mediates the CCR2-dependent modulation of the immune environment. However, this does not preclude that other CCR2 ligands may serve a similar role in other systems.

CCL2 has been shown to enhance tumor growth in various cancers, including prostate (Li et al., 2009), breast (Soria et al., 2008), and lung cancer (Cai et al., 2009), and to be elevated in the lungs of NSCLC patients (Arenberg et al., 2000, Rivas-Fuentes et al., 2015). CCL2 has also been shown to mediate its tumor-supportive ability by acting as a potent chemoattractant for MDSCs (Fujita et al., 2011) and unpolarized monocytes while contributing to polarization of monocytes to MDSCs by increasing their CD206 expression (Roca et al., 2009). As supernatants of TRAIL-treated cells were also able to polarize human CD14^+^ cells toward HLA-DR^lo/neg^ CD206^+^ cells in vitro, it is conceivable that TRAIL-induced cytokines may not only recruit myeloid cells to, but also promote their polarization within, the tumor microenvironment.

Although TRAIL’s immunomodulatory effects could be attributed to a TRAIL/TRAIL-R–CCL2/CCR2 axis, it is important to note that TRAIL’s cancer secretome includes several other cytokines associated with tumor-supportive functions. Therefore, antagonizing TRAIL may be a therapeutic strategy to consider for simultaneously blocking a wider protumorigenic cytokine network as opposed to therapeutic blockade of single constituents thereof.

Our study establishes tumor cell-expressed TRAIL-R as a trigger for cancer cells to secrete CCL2, which, in turn, drives accumulation of a pro-tumorigenic immune microenvironment via host cell-expressed CCR2. In addition to revealing a previously unknown facet of the TRAIL system in tumor biology, the discovered link between endogenous TRAIL/TRAIL-R signaling and a tumor-supportive immune microenvironment suggests that inhibiting the interaction of TRAIL with its receptors might serve as an effective therapeutic option to limit the presence of tumor-supportive myeloid cells within the tumor microenvironment.

## STAR★Methods

### Key Resources Table

**Table:** 

REAGENT or RESOURCE	SOURCE	IDENTIFIER
**Antibodies**		

Western Blot		
α-FADD	BD Bioscience	Cat#556402; RRID: AB_396409
α-β-Actin	Sigma	Cat#A1978; RRID: AB_476692
α-TRAIL-R2	Cell Signaling	Cat#3696
α-caspase-8	Scaffidi et al., 1997	N/A
α-TRAIL-R1	ProSci	Cat#PSC-1139-C100
α-RIP-1	BD Bioscience	Cat#610458; RRID: AB_397831
α-cFLIP	Adipogen	Cat#AG-20B-0056-C050
Flow cytometry		
α -TRAIL-R1 (HS101)	Adipogen	Cat#AG-20B-0022-C100
α -TRAIL-R2 (HS201)	Adipogen	Cat#AG-20B-0023-C100
α -TRAIL-R3 (HS301)	Adipogen	Cat#AG-20B-0024-C100
α -TRAIL-R4 (HS402)	Adipogen	Cat#AG-20B-0025-C100
α -CD45-AF700 (murine)	BioLegend	Cat#103127
α -CD11b-PerCP (murine)	BioLegend	Cat#101230
α -GR-1-PE-Cy7 (murine)	BioLegend	Cat#108416
α -CD274-PE (murine)	eBioscience	Cat#12-5982-82; RRID: AB_466089
α -CD206-FITC (murine)	BioLegend	Cat#141703
α -CD11c-BV711 (murine)	BioLegend	Cat#117349
α -F4/80-BV785 (murine)	BioLegend	Cat#123141
α -MHCII-BV510 (murine)	BioLegend	Cat#107635
α -Ly6C-BV450 (murine)	BD Bioscience	Cat#560594; RRID: AB_1727559
α -CD14-APC (human)	BD Bioscience	Cat#555397
α -HLA-DR-FITC (human)	BD Bioscience	Cat#556643
α -HLA-DR-APC-H7 (human)	BD Bioscience	Cat#641393
α -CD206-PerCP-Cy5.5 (human)	BioLegend	Cat#321121
α -CD11b-PE (human)	BD Bioscience	Cat#555388

**Chemicals, Peptides, and Recombinant Proteins**		

iz-TRAIL	Ganten et al., 2006	N/A
FLAG-TRAIL	Ganten et al., 2006	N/A
D-Luciferin, firefly (in vivo)	GoldBIO	Cat#LUCK-1G
RBC lysis buffer	Cambridge Bioscience	Cat#420301
fixable viability dye eFluor780	eBioscience	Cat#65-0865-14
Fc block	BioLegend	Cat#422302

**Critical Commercial Assays**		

Human Cytokine Array kit	R&D Systems	Cat#ARY005
Human CCL2/MCP-1 DuoSet ELISA	R&D Systems	Cat#DY279
Human CXCL1/GRO alpha DuoSet ELISA	R&D Systems	Cat#DY275
Human CXCL8/IL-8 DuoSet ELISA	R&D Systems	Cat#DY208
Human CXCL5/ENA-78 DuoSet ELISA	R&D Systems	Cat#DY254
Mouse CCL2/JE/MCP-1 DuoSet	R&D Systems	Cat#DY479
Mouse CCL5/RANTES DuoSet	R&D Systems	Cat#DY478
Mouse CXCL1/KC DuoSet ELISA	R&D Systems	Cat#DY453
CellTiter-Glo	Promega	Cat#G7572
Cell proliferation assay kit	Millipore	Cat#2750
anti-CD14 coated microbeads	Milteny Biotec	Cat#130-050-201
RNeasy mini spin kit	QIAGEN	Cat#74104
RevertAid First Strand cDNA synthesis kit	Thermo Fisher	Cat#K1632

**Deposited Data**		

The TRAIL-induced cancer secretome	This paper	ProteomeXchange: PXD005664

**Experimental Models: Cell Lines**		

A549-luc	Caliper Life Science	Bioware Cell Line A549-Luc-C8
3LL	Provided by S. Quezada	N/A
Colo357	Provided by A. Trauzold	N/A
HCT116 WT	Provided by B. Vogelstein	N/A
HCT116 Bax/Bak KO	Provided by B. Vogelstein	N/A
HeLa	Cell stock	N/A

**Experimental Models: Organisms/Strains**		

Mice (C57BL/6)	Charles River UK	C57BL/6NCrl
Mice (Fox Chase SCID Beige)	Charles River UK	CB17.Cg-PrkdcscidLystbg-J/Crl

**Recombinant DNA**		

MSCV-IRES-GFP	Provided by V. Horejsi	N/A

**Sequence-Based Reagents**		

MISSION pLKO.1-puro Empty Vector Control Plasmid DNA	Sigma	Cat#SHC001
mTRAIL-R shRNA set	Sigma	Cat#SHCLND-NM_020275
ON-TARGETplus RIPK1 siRNA smart pool	GE Dharmacon	Cat#L-004445-00-0005
ON-TARGETplus Mouse Tradd (71609) siRNA - SMARTpool	GE Dharmacon	Cat#L-061669-00-0005
ON-TARGETplus Mouse Casp8 (12370) siRNA - SMARTpool	GE Dharmacon	Cat#L-043044-00-0005
ON-TARGETplus Human TRADD (8717) siRNA - SMARTpool	GE Dharmacon	Cat#L-004452-00-0005
ON-TARGETplus Human MAP3K7 (6885) siRNA - SMARTpool	GE Dharmacon	Cat#L-003790-00-0005
siGENOME Non-Targeting siRNA #1	GE Dharmacon	Cat#D-001210-01-05
siGENOME Human CASP8 (841) siRNA - Set of 4 Upgrade	GE Dharmacon	Cat# MU-003466-05-0005
ON-TARGETplus Human BIRC3 siRNA - SMARTpool	GE Dharmacon	Cat# L-004099-00-0005
ON-TARGETplus Human BIRC2 (329) siRNA - SMARTpool	GE Dharmacon	Cat# L-004390-00-0005

**Software and Algorithms**		

MaxQuant version 1.4.1.2	N/A	N/A
MSstats converter script	http://msstats.org/	N/A
Phobius	http://phobius.sbc.su.se/	N/A
DAVID Bioinformatics Resources 6.7	https://david.ncifcrf.gov/	N/A
limma R package	https://bioconductor.org/packages/release/bioc/html/limma.html	N/A

### Contact for Reagent and Resource Sharing

Further information and requests for reagents may be directed to the Lead Contact, Prof. Henning Walczak (h.walczak@ucl.ac.uk).

### Experimental Model and Subject Details

All animal experiments were conducted under an appropriate animal project license approved by the UK home office and held by H. Walczak, in accordance with the revised (2013) Animals (Scientific Procedures) Act (ASPA) and the institutional guidelines of the UCL Cancer Institute. The required risk assessments were obtained for this study. All healthy human donors gave their approval for use of their blood for this research.

### Method Details

#### A549-luc lung cancer xenograft

6-8 week-old female SCID beige mice were obtained from Charles River (UK) and injected with 2 × 10^6^ A549-luc control or FADD knockout cells into the lateral tail vein. 30 min after cell injection, mice were injected with Luciferin (GoldBIO) and imaged for luciferase activity using the Xenogen system to verify presence of cells in the lung. Imaging was repeated weekly for 3 weeks. Animals were euthanized and lungs analyzed for immune infiltrates and by histology.

#### 3LL syngeneic lung cancer model

6-8 week-old C57BL/6 mice of mixed genders were obtained from Charles River (UK) and injected with 5 × 10^5^ 3LL cells stably expressing pLKO.1 control or an shRNA against mTRAIL-R as well as a GFP reporter construct. CCR2 KO mice, on C57BL/6 background, were kindly provided by D. Gilroy. Tumors were left to develop for 4 weeks followed by immune-cell analysis and histology.

#### Histology

Lungs were fixed in 10% Formalin (Sigma), for 48 hr, paraffin-embedded and cut into 5 μm sections. Subsequently, paraffin sections were H&E-stained. Tumor burden was quantified as % lung covered by tumor tissue as determined by an experienced pathologist in a blinded manner.

#### Flow Cytometry

Lungs were cut into small pieces followed by passage through a 40uM Nylon filter (BD). Red blood cells were lysed for 5 min at room temperature (RT) in RBC lysis buffer (BioLegend). Cells were then labeled with fixable viability dye eFluor780 (eBioscience) for 30 min in the dark at RT, followed by Fc block (BD Biosciences). Antibodies used were against CD45-AF700, CD11b-PercpCy5.5, CCR2-AlexaFluor647, GR-1-PE-Cy7, CD274-PE, CD206-FITC, CD11c-BV711, F4-80-BV785, MHCII-BV510, Ly6C-BV450 (detailed in the Key Resources Table). Human monocyte-derived macrophages and MDSC were stained with antibodies against CD14-APC, HLA-DR-FITC, HLA-DR-APC-H7, CD206-PE-Cy5, CD11b-PE (Key Resources Table). Intracellular staining was performed using an Intracellular Fixation and Permeabilization Buffer Set (eBioscience) according to the manufacturer’s instructions. Fluorescence minus one (FMO) controls were used to distinguish between positively and negatively stained cells. Flow cytometric analysis was performed with a LSRFORTESSA X-20 (BD) or Accuri (BD) with subsequent data analysis using FlowJo software.

#### Cell culture and lentiviral infection

HeLa, 3LL and HCT116 cells were cultured in DMEM supplemented with 10% FCS, COLO357 in RPMI1640 supplemented with 10% FCS, 2 mM Glutamine and 1 mM sodium pyruvate and A549-luc cells in RPMI1640 supplemented with 10% FCS. All cell lines were mycoplasma-free as determined by *MycoAlert* Mycoplasma Detection Kit (LONZA). To generate shTRAIL-R 3LL cells, parental 3LL cells were infected with pLKO.1 vector control or shRNA, against TRAIL-R, carrying lentiviral particles for 48 hr. Both vector and shRNA were purchased from Sigma. After selection with 12ug/ml Puromycin, KD efficiency was verified by western blot.

#### RNA interference

Cells were transiently transfected with siRNA pools (ON-TARGET plus or siGENOME) containing four different siRNA sequences targeting each gene of interest or Non-targeting control siRNA. All siRNA pools were purchased from Dharmacon/Thermo Scientific (Loughborough, UK). Cells were transfected using Dharmafect reagent according to the manufacturer’s instructions. Cells were used for further analysis at 48 or 72 hr after transfection as indicated in the figure legends. KD efficiency was confirmed by western blotting.

#### Production of knockout cell lines

A549 FADD KO were generated via zinc finger nucleases (Sigma) targeting exon 1 of FADD. Cells were transfected using Lipofectamine 2000 according to manufacturer instructions. Limiting dilution was employed to achieve single cell cloning, which was followed by KO validation via western blot.

3LL FADD KO were generated via clustered regularly interspaced short palindromic repeats (CRISPR)-Cas-9 technology by targeting exon 2 of murine FADD. The cells were transfected, subjected to single cell cloning and validated as above.

#### Retroviral transduction of cells

WT FADD was inserted into the retroviral MSCV vector, followed by an internal ribosome entry site (IRES) and the open reading frame of EGFP. Lipofectamine 2000 was used to transfect the vector construct into Phoenix cells. The medium was changed 24h post-transfection followed by collection of viral supernatants after 72h. The viral supernatants were then filtered through a 0.45 μm filter and added to the cells in presence of polybrene (6 μg/ml) followed by spinfection (2500 rpm, 45min, 30°C). EGFP positive single cells were then sorted into 96w plates using BDAria.

#### Lysates, Western Blots and antibodies

Cells were lysed in IP-lysis buffer (30 mM Tris-HCl [pH 7.4], 120 mM NaCl, 2 mM EDTA, 2 mM KCl, 1% Triton X-100, 1 × COMPLETE protease-inhibitor cocktail) at 4°C for 30 min. Proteins were separated by SDS-PAGE (NuPAGE) and analyzed by western blotting. Membranes were stripped with 50 mM glycine (pH 2.3) before reprobing with other antibodies (detailed in the Key Resources Table).

#### ELISA and Cytokine arrays

The respective cells were stimulated with iz-TRAIL [100ng/ml]. After 24h, cells were spun at 1500rpm for 3min followed by removal of supernatants. Cytokine levels in the cell supernatants were determined via ELISAs or human cytokine array, according to the manufacturer’s instructions (R&D systems).

#### RNA isolation, cDNA synthesis, and real-time qPCR analysis

Total RNA was extracted from cells or tissues (QIAGEN kit), treated with DNase1 (Life technologies) and reverse transcribed using the RevertAid First Strand cDNA synthesis kit (Fermentas). qPCR synthesis reactions were performed in 96-well plates with 50ng of cDNA on a Realplex Mastercycler (Eppendorf). Relative mRNA levels were calculated using the ΔΔCt method normalized to GAPDH mRNA.

#### Viability and Cell proliferation assays

Cell viability was determined by CellTiter-Glo assay (Promega) according to the manufacturer’s instructions. Cell proliferation was determined by BrdU incorporation (Cell proliferation assay kit, Millipore) according to the manufacturer’s instructions.

#### Luciferase Assay

Stable A549-luc cells were seeded in 96-well plates at the indicated numbers. 24h later, medium was removed and the cells were incubated with 30 μl of Permeabilization Buffer (eBioscience) for 15 min. Subsequently, the cells were incubated with 30 μl Firefly luciferin-containing buffer (Luciferase Assay buffer) for 10 min. A Mithras plate reader was used to determine relative luminescence.

#### Monocyte differentiation

A549 supernatants were generated by stimulating with iz-TRAIL in RPMI1640 for 2h, followed by 2 washes with PBS and centrifugation at 5000rpm for 5min. PBMCs from healthy adult donors were isolated via Ficoll density gradient centrifugation. CD14^+^ monocyte isolation was conducted using anti-CD14 coated microbeads (Milteny Biotec), followed by purity analysis via flow cytometry. Monocytes were cultured at 1x10^6^ cells/ml in A549 supernatants supplemented with 10% human serum; 48h later the differentiation state was determined via flow cytometry.

#### Sample preparation for mass spectrometry

Proteins were denatured with 6M urea in 50mM ammonium bicarbonate (AB). Denatured proteins were reduced with 4mM dithiothreitol (DTT) in 50mM AB at 56°C for 25min, cooled to room temperature and alkylated with 8mM iodoacetamide in 50mM AB at room temperature in the dark for 30min. The excess of iodoacetamide was removed with an additional 4mM DTT in 50mM AB. Urea concentration was then diluted to 1.5M with 50mM AB. Proteins were digested with sequencing grade trypsin (Promega) at a 1:50 protease to protein ratio at 37°C for 15h. Digestion was stopped with 1% trifluoroacetic acid (TFA). Peptide digests were desalted with microspin columns filled with SEM. SS18V silica (The Nest Group), eluted with 50% acetonitrile 0.1% TFA, evaporated to dryness at 30°C, and resolubilized in 20μL of 10% formic acid in water. 1-2μL of peptides was used for nLC-MS analysis.

#### Mass spectrometry

nLC-MS/MS was performed on a Q Exactive Orbitrap interfaced to a NANOSPRAY FLEX ion source and coupled to an Easy-nLC 1000 (Thermo Scientific). Peptides were separated on a 20cm fused silica emitter, 75μm diameter, packed in-house with Reprosil-Pur 200 C18-AQ, 2.4μm resin (Dr.Maisch) using a linear gradient from 5% to 30% acetonitrile/ 0.1% formic acid over 4h, at a flow rate of 300 nL/min. Precursor ions were measured in a data-dependent mode in the orbitrap analyzer at a resolution of 70,000 and a target value of 1e6 ions. The ten most intense ions from each MS1 scan were isolated, fragmented in the HCD cell, and measured in the orbitrap at a resolution of 17,500. The total protein content of the concentrated secretome was determined with the Bicinchoninic acid (BCA) assay. An equal protein load was used across conditions, which was in the range of 10-100ug between replicate experiments.

### Quantification and Statistical Analysis

#### In vivo bioluminescence quantification

Photon flux was determined by Xenogen software quantifying photons per second in a defined region of interest (ROI).

#### Immune cell quantification by Flow cytometry

Flow cytometric reference beads (Invitrogen) were mixed to the samples before analysis for quantification of immune cell subsets in each lung. The absolute number (Abs) of cells was calculated using the following formula: Abs = (count of gated cells/count of beads)/number of beads added to the sample.

#### Statistics

Data were analyzed using the GraphPad Prism 6 software (GraphPad Software). Results are means ± SEM. Statistical significance between groups was determined using Student’s t test. A p value of < 0.05 was considered statistically significant and indicated with ^∗^, p < 0.01 = ^∗∗^ and p < 0.001 = ^∗∗∗^. ns = non-significant.

#### Mass spectrometric data analysis

Raw data were analyzed with MaxQuant version 1.4.1.2 where they were searched against the human UniProt database (http://www.uniprot.org/, downloaded 03/04/2013) using default settings. Carbamidomethylation of cysteines was set as fixed modification, and oxidation of methionines and acetylation at N-termini were set as variable modifications. Enzyme specificity was set to trypsin with maximally 2 missed cleavages allowed. To ensure high confidence identifications, PSMs, peptides, and proteins were filtered at a less than 1% false discovery rate (FDR). The label-free quantification workflow was used with a match time window of 4min, an alignment time window of 20mins, and a match between runs selected. Statistical protein analysis was performed with MSstats.daily_2.3.4 (Choi et al., 2014). MaxQuant output was converted to the MSstats required format with a converter script from the MSstats website (http://msstats.org/). p values were adjusted to control for the false discovery rate using the Benjamini-Hochberg procedure (Benjamini and Hochberg, 1995). Quantified proteins were annotated with cellular compartments reported in UniProt and predicted with the transmembrane topology and signal peptide predictor Phobius (http://phobius.sbc.su.se/). Identified intracellular contaminants were removed, and secreted and membrane proteins were retained to comprise the secretome. Upregulated secretome proteins by at least two fold upon TRAIL treatment were submitted to DAVID Bioinformatics Resources 6.7 for enrichment analysis of gene ontology molecular function terms. The quantification of proteins associated with the top three enriched molecular functions (chemokine activity, chemokine receptor binding, cytokine activity) were visually inspected and in cases where noisy peptide features were present (standard error > 0.3) or where peptide features were not extracted in all replicates by MaxQuant, the peptide with the highest signal to noise ratio for that protein was reanalyzed in Skyline (MacLean et al., 2010) by targeted data quantification. A student’s paired t test with a one-tail distribution was used for the comparisons of means.

#### TCGA expression analysis

RNaseq V2 level 3 data were downloaded for 489 LUAD samples from the TCGA data portal and parsed using a custom R function. The RSEM expression values were transformed to log2 counts per million using the voom function from the limma R package. High and Low composite groups for TRAIL & CCL2, or TRAIL & CCR2, were defined using overlapping samples for both genes of a pair in the top and bottom 50% of expression values. Differentially expressed genes were determined using limma-trend at a BH-adjusted p value of 0.01 and a two-fold change in expression between the composite high and low groups and were filtered to a curated list of immune factors for visualization on heatmaps.

### Data and Software Availability

Expression data from the LUAD patient cohort were downloaded from the Cancer Genome Atlas (TCGA) Research Network and can be obtained at https://cancergenome.nih.gov/. The accession number for the “TRAIL-induced cancer secretome” dataset reported in this paper is ProteomeXchange: PXD5664 and can be obtained at http://www.ebi.ac.uk/pride.

## Author Contributions

H.W. conceived the project. T.H., A.M., S.v.K., S.A.Q., and H.W. designed the research and wrote the manuscript. T.H. and A.M. performed the majority of the experiments. S.v.K. assisted with the in vivo experiments; A.S. helped in designing the in vitro polarization experiments. S.S. performed the mass spectrometric analysis of the secretome and analyzed the resulting data. A.C. performed the bioinformatic analysis. A.S., L.T., P.D., E.L., and F.A.V. contributed experimentally. M.A.E.-B. performed the histopathological analysis of lung tumor tissues in a blinded manner.

## Supplemental Information

Document S1. Figures S1–S7 and Table S2

Table S1. The TRAIL-Induced Secretome, Related to Figure 1

Document S2. Article plus Supplemental Information
