# Supplementary material for: The TRAIL-Induced Cancer Secretome Promotes a Tumor-Supportive Immune Microenvironment via CCR2
Source: Mol Cell. 2017 Feb 16;65(4):730–742.e5. doi: 10.1016/j.molcel.2017.01.021 (PMC5316415; doi:10.1016/j.molcel.2017.01.021)
Supplement: Document S1. Figures S1–S7 and Table S2 [file mmc1.pdf]

Molecular Cell, Volume *65*

## Supplemental Information

# The TRAIL-Induced Cancer Secretome Promotes a Tumor-Supportive Immune Microenvironment via CCR2

**Torsten Hartwig, Antonella Montinaro, Silvia von Karstedt, Alexandra Sevko, Silvia Surinova, Ankur Chakravarthy, Lucia Taraborrelli, Peter Draber, Elodie Lafont, Frederick Arce Vargas, Mona A. El-Bahrawy, Sergio A. Quezada, and Henning Walczak**

# Figure S1

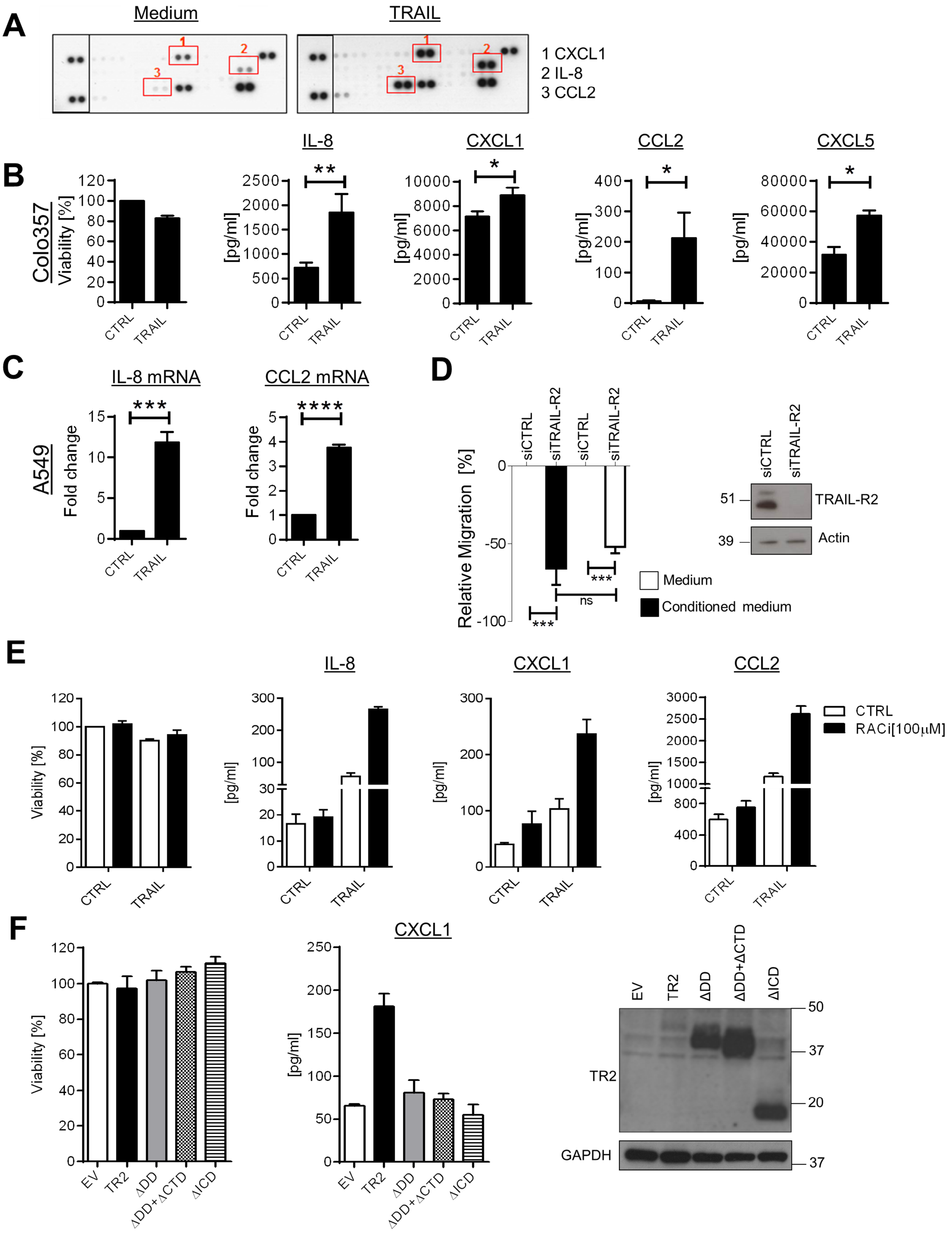
A
Medium
TRAIL
1 CXCL1
2 IL-8
3 CCL2
B
Colo357
Viability [%]
IL-8
CXCL1
CCL2
CXCL5
[pg/ml]
CTRL
TRAIL
C
A549
IL-8 mRNA
CCL2 mRNA
Fold change
D
Relative Migration [%]
siCTRL
siTRAIL-R2
Medium
Conditioned medium
TRAIL-R2
Actin
E
RACi[100μM]
F
EV
TR2
ΔDD
ΔDD+ΔCTD
ΔICD
TR2
GAPDH

Figure S1. Related to Figure 1. TRAIL induces cytokines in various cell lines and requires the DD of TRAIL-R2 for cytokine induction in A549. (A) A549 cells were stimulated for 24h as indicated and supernatants were used for cytokine array (R&D Systems). TRAIL-induced cytokines are highlighted in red. (B) The indicated cell lines were stimulated with iz-TRAIL [100 ng/ml] for 24h; cell viability was determined by CellTiter-Glo and supernatants were subjected to the indicated ELISAs. (C) A549 cells were stimulated as in (B), RNA was extracted and mRNA levels for IL-8 and CCL2 were determined by qPCR. (D) A549 cells with the indicated knockdowns were subjected to migration assays described previously (von Karstedt et al., 2015) in the presence of either medium control or A549-conditioned medium in the upper transwell chamber. (E) A549 cells were treated with iz-TRAIL [100 ng/ml] for 24h in the presence or absence of Rac1 inhibitor (NSC23766 [100µM]); cell viability was determined by CellTiter-Glo and the indicated cytokines were quantified from supernatants by ELISA. (F) A549 cells were transiently transfected with either empty vector (EV) or with vector containing the indicated TRAIL-R2 (TR2) version and subsequently subjected to CellTiter-Glo; supernatants from these cells were subjected to CXCL1 ELISA. A representative western blot of cells in (F) is shown. Unpaired, two-tailed Student's t test was performed to determine significance. *$P \leq 0.05$; **$P < 0.01$; ***$P < 0.001$; P****< 0.0001. Data are presented as mean ± SEM. n=3.

# Figure S2

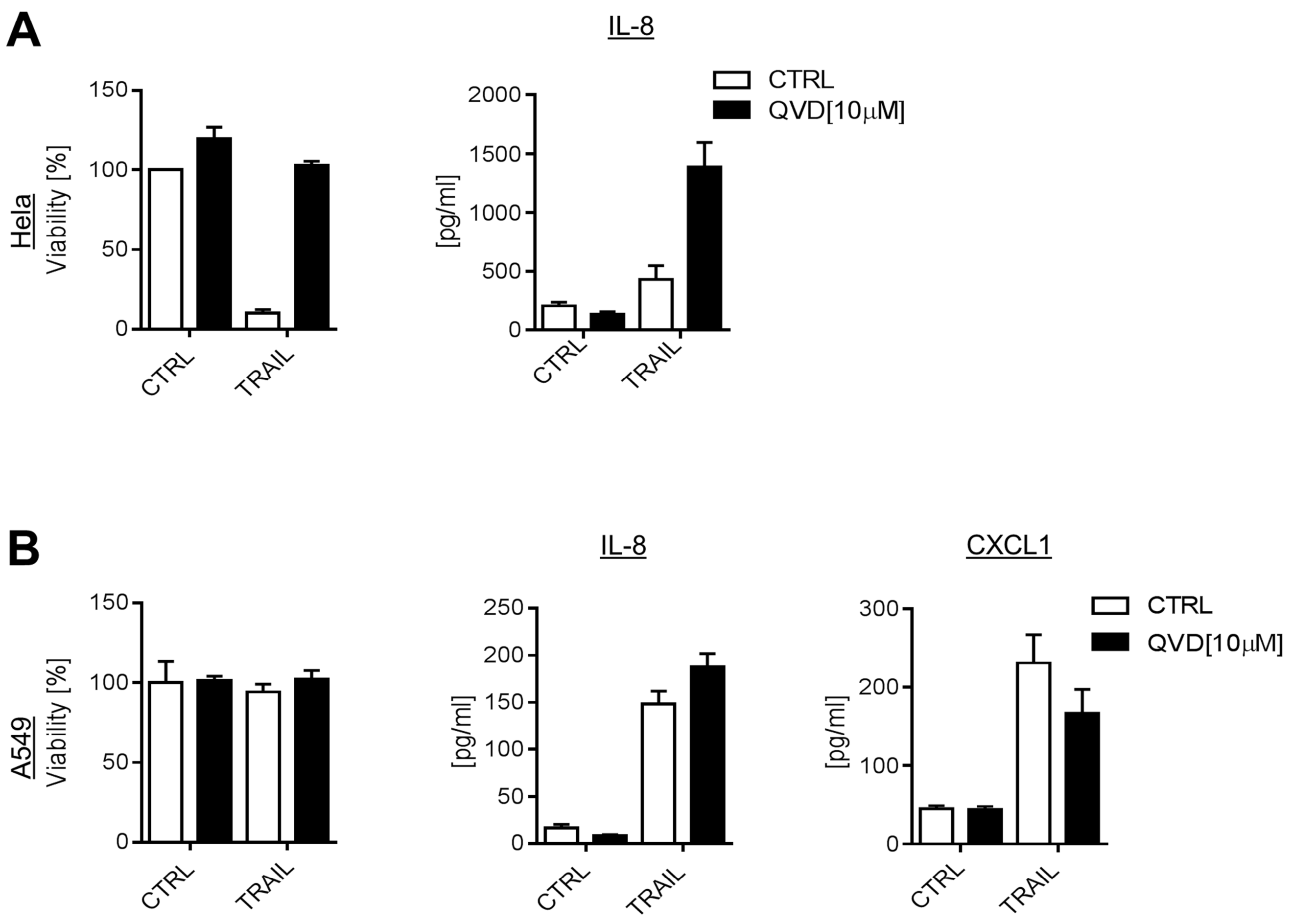


Figure S2. Related to Figure 2. Caspase-8 inhibition rescues cell survival and cytokine production. (A) HeLa cells were pre-incubated with QVD [10µM] or DMSO for 30 min followed by addition of iz-TRAIL [100ng/ml] for 24h; cell viability was determined by CellTiter-Glo and IL-8 concentrations in the cell supernatants were measured by ELISA. (B) A549 cells were treated as in (A) and the indicated cytokines were quantified by ELISA. Data are presented as mean ± SEM. n=3.

# Figure S3

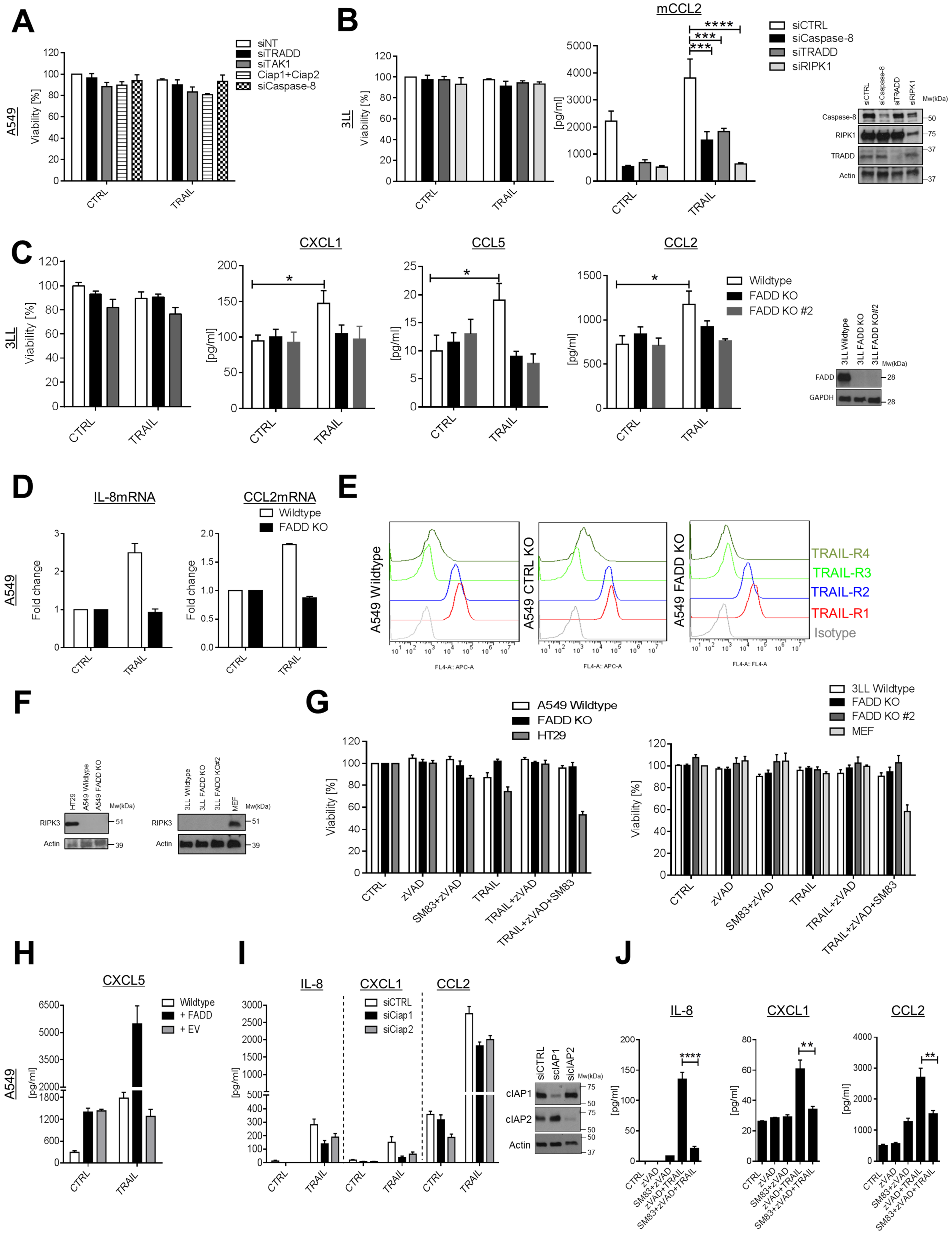
A
A549
Viability [%]
siNT
siTRADD
siTAK1
Ciap1+Ciap2
siCaspase-8
CTRL
TRAIL
B
3LL
Viability [%]
mCCL2
[pg/ml]
siCTRL
siCaspase-8
siTRADD
siRIPK1
Caspase-8
RIPK1
TRADD
Actin
Mw(kDa)
C
3LL
Viability [%]
CXCL1
CCL5
CCL2
[pg/ml]
Wildtype
FADD KO
FADD KO #2
3LL Wildtype
3LL FADD KO
3LL FADD KO#2
FADD
GAPDH
D
A549
IL-8mRNA
CCL2mRNA
Fold change
Wildtype
FADD KO
E
A549 Wildtype
A549 CTRL KO
A549 FADD KO
TRAIL-R4
TRAIL-R3
TRAIL-R2
TRAIL-R1
Isotype
FL4-A:: APC-A
FL4-A:: FL4-A
F
HT29
A549 Wildtype
A549 FADD KO
3LL Wildtype
3LL FADD KO
3LL FADD KO#2
MEF
RIPK3
Actin
G
A549 Wildtype
FADD KO
HT29
3LL Wildtype
FADD KO
FADD KO #2
MEF
Viability [%]
CTRL
zVAD
SM83+zVAD
TRAIL
TRAIL+zVAD
TRAIL+zVAD+SM83
H
A549
CXCL5
[pg/ml]
Wildtype
+ FADD
+ EV
I
IL-8
CXCL1
CCL2
[pg/ml]
siCTRL
siCiap1
siCiap2
sIAP1
sicIAP2
cIAP1
cIAP2
Actin
J
IL-8
CXCL1
CCL2
[pg/ml]
CTRL
zVAD
SM83+zVAD
zVAD+TRAIL
SM83+zVAD+TRAIL

Figure S3. Related to Figure 3. FADD-deficiency abrogates TRAIL-induced cytokine production. (A) A549 cells were subjected to the indicated knockdowns for 48h followed by QVD[10uM](CTRL) or QVD[10uM] + iz-TRAIL treatment [100ng/ml] for another 24h and cell viability was determined by CellTiter Glo. (B) 3LL cells were subjected to the indicated knockdowns for 48h followed by QVD[10uM](CTRL) or QVD[10uM] + iz-mTRAIL treatment [1µg/ml] for another 24h. Cell viability was determined by CellTiter-Glo and supernatant was quantified for CCL2 by ELISA; a representative Western blot is shown. (C) 3LL wildtype or two FADD KO clones were stimulated with iz-mTRAIL [1µg/ml] for 24h. The indicated cytokines were quantified by ELISA. (D) A549 wildtype or FADD KO cells were stimulated with iz-TRAIL [100ng/ml]; after 24h, RNA was extracted and mRNA levels for IL-8 and CCL2 were determined by qPCR. (E) Human TRAIL-R1-4 were stained on the indicated A549 cells and expression levels were determined by FACS. Representative histograms of three independent experiments are shown. (F) The indicated cell lines were probed for RIPK3 expression by Western Blot. (G) A549 and 3LL wildtype or FADD KO clones, in comparison to HT29 or MEFs respectively, were treated with zVAD [10uM], SM-083[100nM] or the combination of both for 24h and subjected viability assays as before. (H) A549 wildtype, or FADD KO either empty vector (+ EV) or FADD (+ FADD) reconstituted were stimulated with iz-TRAIL [100ng/ml] for 24h followed by determination of cytokine concentrations by ELISA. (I) A549 cells were subjected to the indicated knockdowns for 48h followed by QVD[10uM](CTRL) or QVD[10uM] + TRAIL treatment [100ng/ml] for another 24h. Cytokines were quantified by ELISA, a representative Western blot is shown. (J) A549 cells were treated with zVAD [10uM], SMAC mimetic SM-83 [100nM] with or without TRAIL[100ng/ml] for 24h; cytokines were determined as before. Data are presented as mean ± SEM. n=3.

# Figure S4

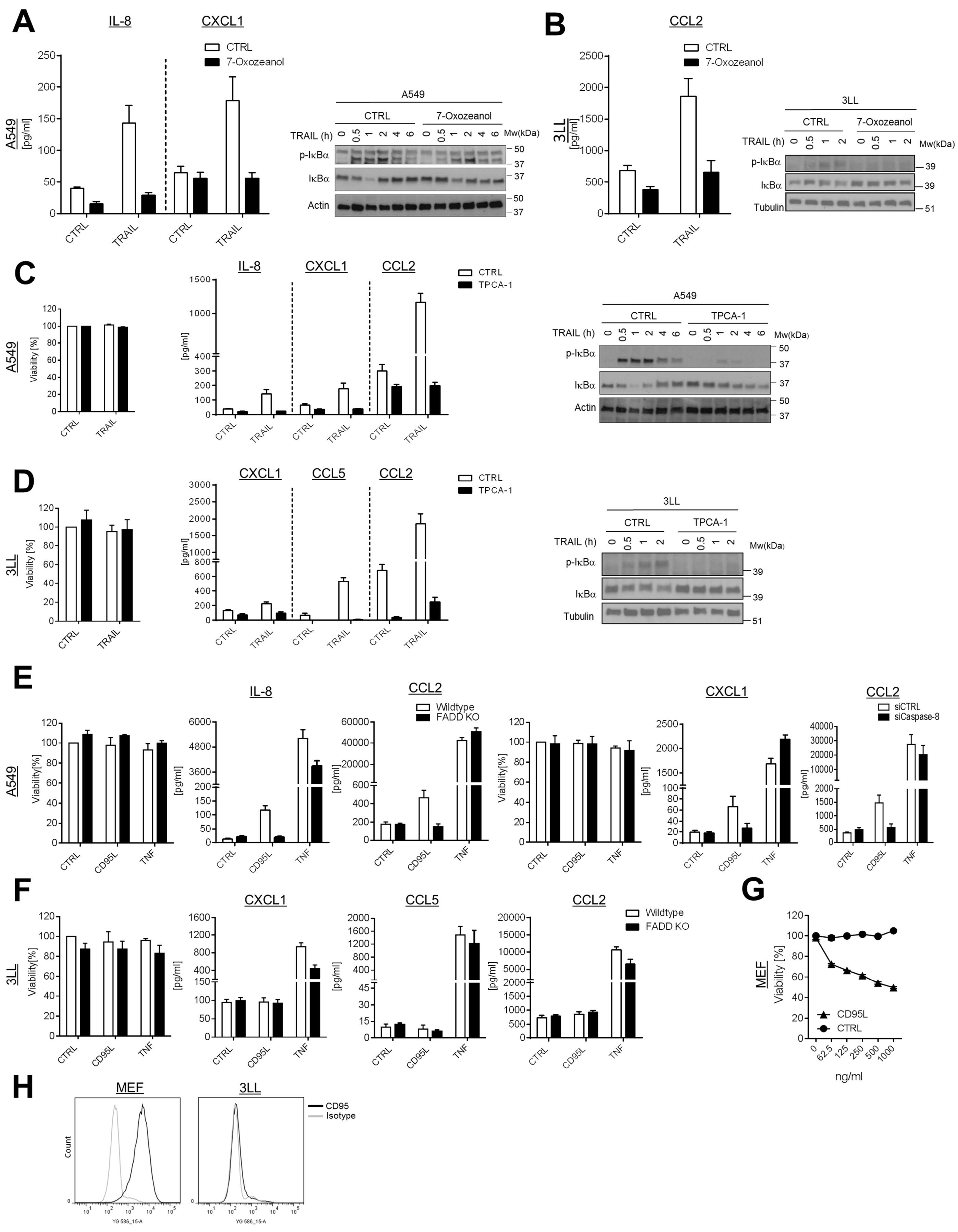

A
IL-8
CXCL1
CTRL
7-Oxozeanol
A549 [pg/ml]
CTRL
TRAIL
A549
CTRL
7-Oxozeanol
TRAIL (h)
Mw(kDa)
p-IκBα
IκBα
Actin
B
CCL2
CTRL
7-Oxozeanol
3LL [pg/ml]
3LL
CTRL
7-Oxozeanol
TRAIL (h)
Mw(kDa)
p-IκBα
IκBα
Tubulin
C
A549
Viability [%]
IL-8
CXCL1
CCL2
CTRL
TPCA-1
[pg/ml]
A549
CTRL
TPCA-1
TRAIL (h)
Mw(kDa)
p-IκBα
IκBα
Actin
D
3LL
Viability [%]
CXCL1
CCL5
CCL2
CTRL
TPCA-1
[pg/ml]
3LL
CTRL
TPCA-1
TRAIL (h)
Mw(kDa)
p-IκBα
IκBα
Tubulin
E
A549
Viability[%]
IL-8
CCL2
Wildtype
FADD KO
[pg/ml]
CXCL1
CCL2
siCTRL
siCaspase-8
CTRL
CD95L
TNF
F
3LL
Viability[%]
CXCL1
CCL5
CCL2
Wildtype
FADD KO
[pg/ml]
G
MEF
Viability [%]
CD95L
CTRL
ng/ml
H
MEF
3LL
CD95
Isotype
Count
YG 586_15-A

Figure S4. Related to Figure 3. Activation of the IKK complex is necessary for TRAIL-induced cytokine production.

(A) A549 cells were treated with 7-Oxozeanol [1uM] and/or TRAIL [100ng/ml] for 24h and indicated cytokines were quantified by ELISA. Cells were stimulated accordingly for the indicated times; a representative western blot is shown. (B) 3LL cells were treated and analysed as in (A). (C) A549 cells were treated with TPCA-1 [5uM] for 24h. Cell viability was determined by CellTiter-Glo and cytokines were quantified by ELISA. A kinetic of TPCA treatment is shown. (D) 3LL cells were treated and analysed as in (C). (E) A549 cells were treated with either TNF [50ng/ml] or CD95L-FC [200ng/ml] for 24h. Cell viability was determined by CellTiter-Glo and cytokines were quantified as before. (F) 3LL cells were treated with TNF [50ng/ml] or CD95L-FC [1µg/ml] for 24h. Cell viability was determined by CellTiter-Glo and cytokines were quantified as before. Data are presented as mean ± SEM. n=3. (G) MEF were stimulated with the indicated concentrations of CD95L-FC for 24h; cell viability was determined by CellTiter-Glo. (H) Mouse CD95 was stained on the indicated cell lines and expression level was determined by FACS. Representative histograms are shown.

# Figure S5

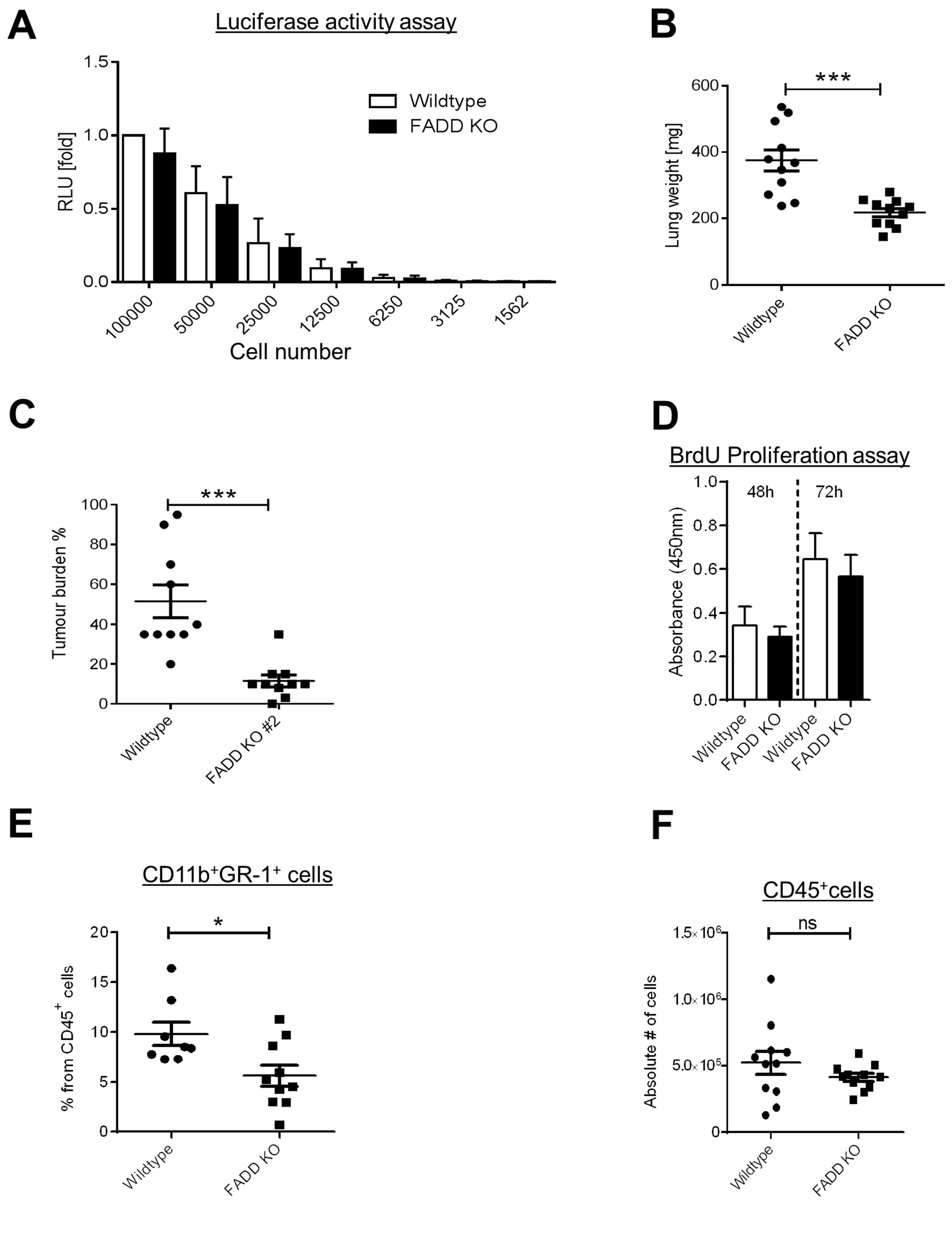
A
Luciferase activity assay
Wildtype
FADD KO
RLU [fold]
1.5
1.0
0.5
0.0
100000
50000
25000
12500
6250
3125
1562
Cell number
B
***
600
400
200
0
Lung weight [mg]
Wildtype
FADD KO
C
***
100
80
60
40
20
0
Tumour burden %
Wildtype
FADD KO #2
D
BrdU Proliferation assay
1.0
0.8
0.6
0.4
0.2
0.0
48h
72h
Absorbance (450nm)
Wildtype
FADD KO
Wildtype
FADD KO
E
CD11b+GR-1+ cells
*
20
15
10
5
0
% from CD45+ cells
Wildtype
FADD KO
F
CD45+cells
ns
1.5×10^6
1.0×10^6
5.0×10^5
0
Absolute # of cells
Wildtype
FADD KO

Figure S5. Related to Figure 4. FADD does not affect in-vitro proliferation or luciferase activity. (A) A549 cells were seeded at the indicated cell numbers and the next day incubated with firefly luciferin. Bioluminescence was quantified using a Mithras plate reader. (B) Lungs containing the indicated A549 cells were weighed. (C) $5 \times 10^5$ 3LL wildtype or a second FADD KO clone were injected via the lateral tail vein. 28 days later tumor burden was determined by pathological inspection from H&E-stained sections. (D) Cells as in (A) CTRL or FADD KO were subjected to proliferation assays by measuring BrdU incorporation after the indicated times. (E) Percentage of CD11b+ GR1+ from $CD45^+$ cells in dissociated 3LL Lungs (F) Absolute numbers of the indicated cells were determined by FACS in dissociated lungs containing A549 WT or FADD KO. Unpaired, two-tailed Student’s t test was performed to determine significance. ns= *P≤ 0.05; ***P < 0.001. Data are presented as mean ± SEM. n=3

# Figure S6

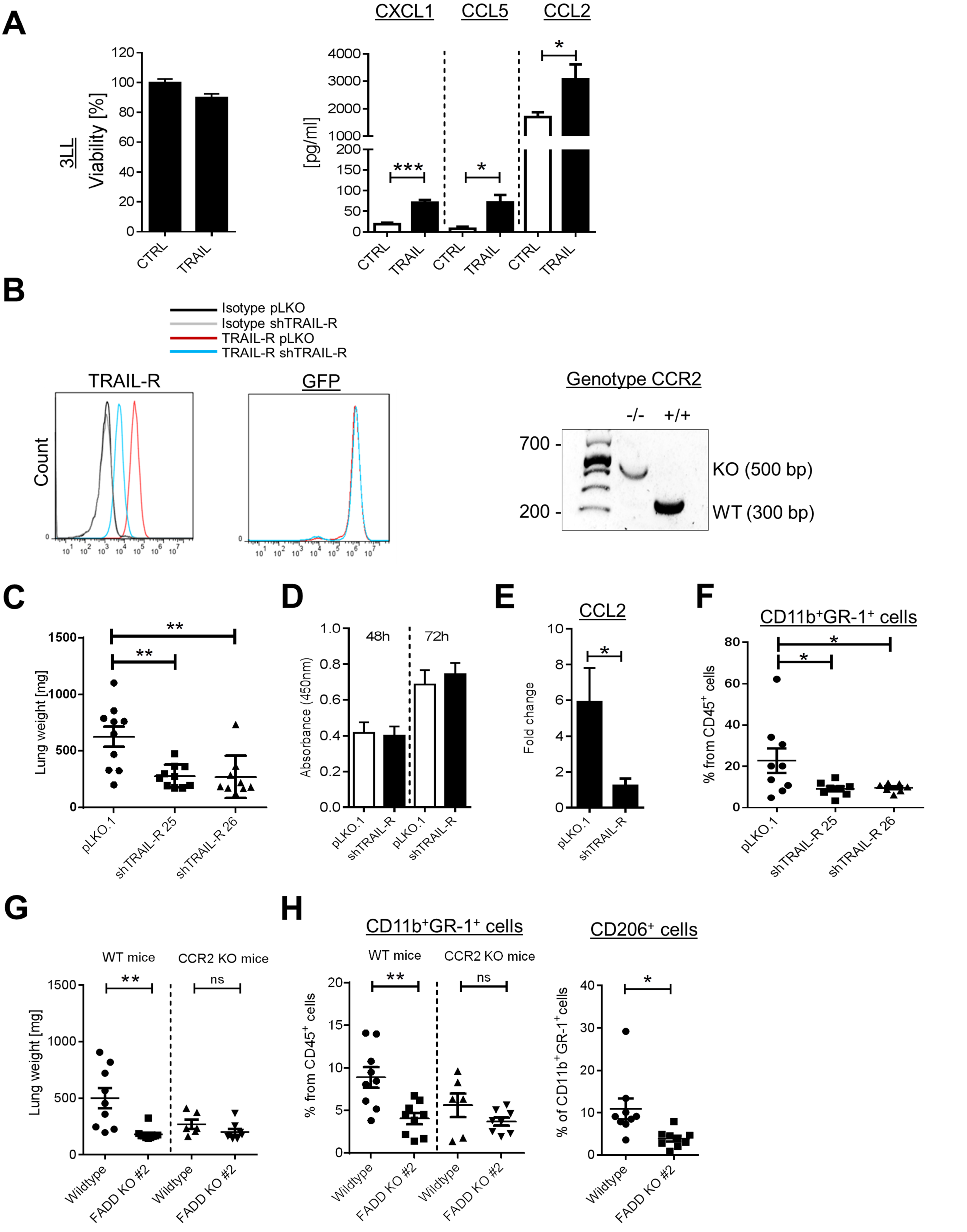


Figure S6. Related to Figure 6. Cancer cell-expressed TRAIL-R supports tumor growth and recruitment of tumor-supportive infiltrates in a host CCR2-dependent manner. (A) 3LL cells were treated with iz-mTRAIL [1µg/ml] for 24h,

viability was determined by CellTiter-Glo and supernatants were subjected to the indicated ELISAs. (B) The indicated 3LL cells were stained with α-TRAIL-R antibody and expression levels and GFP were quantified by FACS (left panel); image of a representative agarose gel showing genotyping results of CCR2 KO and WT animals (right panel). (C) Lung weights of mice injected with 3LL cells containing the indicated shTRAIL-R constructs. (D) 3LL containing pLKO.1 or shTRAIL-R constructs were subjected to BrdU incorporation for the indicated times. (E) Fold change in CCL2 mRNA expression in WT lungs containing plKO.1 or shmTR cells as determined by qPCR. (F) $CD11b^{+}GR\text{-}1^{+}$ infiltrates corresponding to lungs in (C) were determined by FACS. (G) Lung weights in wildtype or CCR2 KO mice containing the indicated cells were determined. (H) Immune infiltrates corresponding to lungs in (G) were determined by FACS. Unpaired, two-tailed Student's t test was performed to determine significance. *$P < 0.05$; **$P < 0.01$ ***$P < 0.001$. Data are presented as mean ± SEM. n=3.

# Figure S7

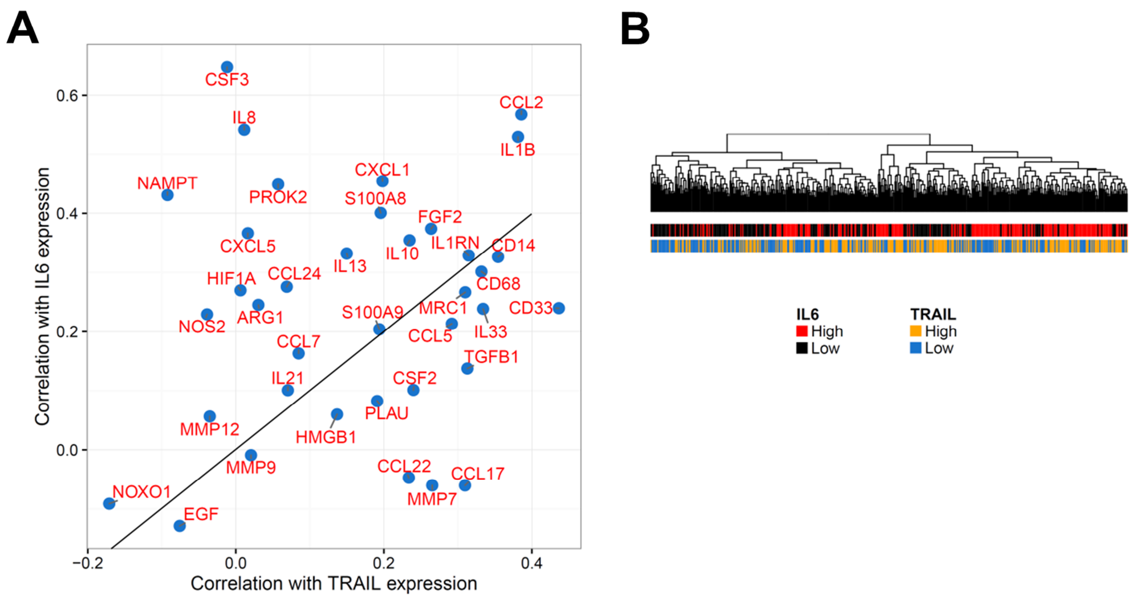


Figure S7. Related to Figure 7. TRAIL and IL-6 correlate with a common cytokine network. (A, B) RNAseq expression data from human lung adenocarcinoma biopsy samples (n=489) were analyzed for association of TRAIL (TNFSF10) or IL-6 expression for a curated list of immune-related genes. (A) Correlation plot of either TRAIL or IL-6 association with the curated list of immune-related genes. (B) Patients were clustered according to 50% high or low TRAIL or IL-6 expression.

Table S1. Related to Figure 1. TRAIL-induced Secretome

See separate Excel file

# Table S2

| |
|---|
| CCL2 |
| CXCL1 |
| IL-8 |
| NAMPT |
| CD11b |
| CD14 |
| CD33 |
| MRC1 |
| CD15 |
| CD68 |
| VEGF |
| Gm-CSF |
| G-CSF |
| PGE2 |
| M-CSF |
| CXC12 |
| S100A8 |
| S100A9 |
| TGFb |
| CXCL5 |
| IL-1beta |
| IL6 |
| IL10 |
| IL12 |
| IL13 |
| IL14 |
| HIF-1alpha |
| HMGB1 |
| MMP9 |
| Bv8 |
| bFGF |
| Nitric oxide synthase (iNOS) |
| NADPHox |
| Arginase-1 (ARG-1) |
| CCL7 |
| CCL5 |
| IL33 |
| IL21 |
| CCL17 |
| CCL22 |
| CCL24 |
| uPA |
| EGF |
| IL1-ra |
| Mmp7 |
| Mmp12 |

Table S2. Related to Figure 7. List of immune markers and cytokines for gene expression analysis
